# Early smoking and its impact on cardio-cerebrovascular diseases in patients with chronic kidney disease: a nationwide population-based study

**DOI:** 10.1186/s12889-025-23276-0

**Published:** 2025-06-03

**Authors:** Sehyun Jung, Kyungdo Han, Seong Geun Kim, Semin Cho, Hyuk Huh, Jung Hun Koh, Jeongmin Cho, Minsang Kim, Min Woo Kang, Eunjeong Kang, Sehoon Park, Yaerim Kim, Kwon Wook Joo, Dong Ki Kim, Soojin Lee

**Affiliations:** 1 Department of Internal Medicine, Gyeongsang National University Hospital, Jinju, Republic of Korea; 2 Department of Statistics and Actuarial Science, Soongsil University, Seoul, Republic of Korea; 3 Department of Internal Medicine, Inje University Sanggye Paik Hospital, Seoul, Republic of Korea; 4 Department of Internal Medicine, Chung-Ang University Gwangmyeong Hospital, Gwangmyeong, Republic of Korea; 5 Department of Internal Medicine, Hallym University Kangnam Sacred Heart Hospital, Seoul, Republic of Korea; 6 Department of Internal Medicine, Seoul National University Hospital, Seoul, Republic of Korea; 7 Department of Internal Medicine, Korea University Guro Hospital, Seoul, Republic of Korea; 8 Department of Transplantation Center, Seoul National University Hospital, Seoul, Republic of Korea; 9 Department of Internal Medicine, Keimyung University School of Medicine, Daegu, Republic of Korea; 10 Department of Internal Medicine, Seoul National University College of Medicine, Seoul, Republic of Korea; 11 Department of Internal Medicine, Uijeongbu Eulji University Medical Center, 712, Dongil-ro, Uijeongbu-si, Gyeonggi-do, 11759 Republic of Korea

**Keywords:** Smoking, Chronic kidney disease, Cardio-cerebrovascular disease

## Abstract

**Objective:**

Smoking is a leading preventable cause of disease and death worldwide, with severe implications for individuals with chronic kidney disease (CKD). Although smoking at a younger age is linked to higher mortality risk, the specific effects of early smoking on all-cause and cardio-cerebrovascular diseases (CCVDs)-specific mortality in CKD patients are not well established. This study aims to examine the association between early smoking, smoking intensity, and mortality in patients with CKD.

**Methods:**

This nationwide, population-based cohort study utilized data from the National Health Insurance Database (NHID) of South Korea, provided by the National Health Insurance Service (NHIS). The study included 549,739 adults with CKD who underwent national health examinations in 2009. The primary exposures were the age at smoking initiation and smoking intensity, measured in pack-years. Cox proportional hazards models were used to analyze the association between these exposures and mortality outcomes.

**Results:**

Earlier smoking initiation and higher smoking intensity were significantly associated with increased risks of all-cause and CCVDs-specific mortality among patients with CKD. Specifically, individuals who began smoking at a younger age and those with higher pack-years had a notably higher risk of mortality.

**Conclusions:**

The findings suggest that early smoking and smoking intensity are associated with higher mortality risks in CKD patients. Preventive measures targeting early smoking initiation may help improve the long-term outcomes in high-risk population.

**Supplementary Information:**

The online version contains supplementary material available at 10.1186/s12889-025-23276-0.

## Introduction

Smoking is a major, preventable cause of numerous diseases and death worldwide [1–4]. Smoking adversely affects multiple organs, and is known to cause cardiovascular complications and various cancers [3, 5, 6]. Previous studies have highlighted the association between age at smoking initiation and mortality rates, and revealed that individuals who started smoking at a younger age showed an elevated risk of mortality [7–10].

Chronic kidney disease (CKD) is associated with an increased risk of cardiovascular and cerebrovascular diseases [11, 12]. Cardiovascular events are the leading cause of death in patients with CKD [13]. Smoking is an important risk factor that accelerates CKD progression [14], increases the risk of cardiovascular complications and mortality in patients with CKD [15]. Moreover, several studies have demonstrated that current smokers with CKD have an increased risk of adverse events, such as vascular events, cancer, and all-cause mortality, compared to never smokers [15, 16].

Thus, smoking cessation and minimizing smoking exposure are crucial for mitigating further adverse events in patients with CKD. To reduce the morbidity and mortality rates, comprehensive public health measures focusing on reducing smoking demand are essential, particularly through preventive efforts targeting early smoking initiation and clinical interventions aimed at promoting smoking cessation among smokers [17].

The aim of the present study was to investigate the adverse effects of early smoking initiation in patients with CKD. We investigated the all-cause and cardio-cerebrovascular disease (CCVDs)-specific mortality rates according to smoking initiation age and smoking intensity in patients with prevalent CKD aged 20 years or older who underwent the national health examination in 2012.

## Materials and methods

### Ethical consideration

This study obtained approval from the Institutional Review Board of Seoul National University Hospital (IRB No. E-2001-112-1096). The use of the National Health Insurance Database (NHID) was approved by a government organization. The study was conducted in accordance with the Declaration of Helsinki.

## Data source

We conducted a nationwide population-based cohort study by reviewing the National Health Insurance Database (NHID) provided by the National Health Insurance Service (NHIS) in South Korea [18].

All citizens of the Republic of Korea are covered by the National Health Insurance. The complimentary general health check-up provided by the NHIS includes measurements of serum creatinine levels and urine stick albuminuria. The NHIS provides complimentary general health checkups annually for nonoffice workers and biannually for office workers or nonworkplace subscribers. Dependent members over the age of 40 years also receive a checkup every two years. Since 2009, the general health checkup rate has been approximately 70% in approximately 15 million eligible people. The NHID provided by the NHIS is an insurance claims database that encompasses information related to national general health checkups, sociodemographic variables, and mortality rates [19]. To ensure data integrity, we used a standardized dataset provided by the National Health Insurance Service (NHIS), which includes verified diagnostic codes, prescription records, and mortality data. Quality control measures were implemented by NHIS, and data preprocessing included logical checks and exclusion of biologically implausible values.

## Study population

**Fig. 1:**
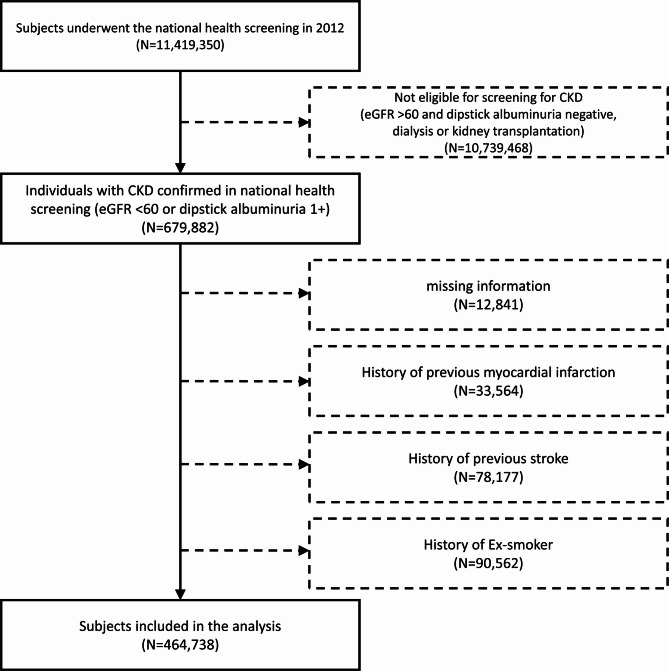
Study flow chart. Flow chart represents the selection of study participants. CKD, chronic kidney disease

A total of 11,419,350 adults aged 20 years and above who received the national health screening in 2012 were considered. Among these, 679,882 individuals were diagnosed with CKD during the 2012 national health screening. CKD was defined as eGFR of less than 60 mL/min/1.73 m^2^ or a positive result on the dipstick albuminuria test in the national health examination. These individuals were regarded as having prevalent CKD at the time of enrollment, as the duration of disease prior to screening could not be determined. Individuals receiving dialysis or those who received kidney transplantation were excluded from the study. To eliminate confounding variables, we excluded 12,841 participants with missing data, 33,564 patients previously diagnosed with myocardial infarction, 78,177 with stroke at baseline, and 90,562 former smokers. Finally, 464,738 participants were included in the study (Fig. 1).

We included participants who underwent health examinations in 2012, and the follow-up period continued until December 31, 2021. The average follow-up duration was 8.99 years. The first, second (median), and third quartiles (Q1/Q2/Q3) of follow-up duration were 9.09/9.35/9.65 years.

## Study exposure

The study exposures were the estimated glomerular filtration rate (eGFR) and dipstick albuminuria measured during national health examinations. Additionally, data, including smoking status, age at smoking initiation, and pack-years smoked, were extracted from the National Health Screening Questionnaire.

## Data collection

The NHID provided the baseline characteristics, including age; sex; low-income status; history of diabetes mellitus, hypertension, and dyslipidemia; alcohol consumption; regular exercise; body mass index; waist circumference; blood pressure; and baseline laboratory parameters, including fasting glucose values, lipid profiles, estimated glomerular filtration rate, and proteinuria from urine tests. Low-income status was defined as an income below the 25th percentile of the country’s income distribution. Baseline comorbidities, including diabetes mellitus, hypertension, and hyperlipidemia, were inferred from the tenth edition of the International Classification of Diseases (ICD-10) diagnostic codes and prescription records of related medications. The coders used the ICD-10 for myocardial infarction (MI), stroke, and death. The eGFR was calculated using the Modification of Diet in Renal Disease (MDRD) equation. Heavy alcohol consumption was defined as consumption of more than 30 g of alcohol per day, and mild alcohol consumption was defined as consumption of 0–30 g of alcohol per day. Regular physical activity was defined as moderate-intensity physical activity for ≥ 5 days or vigorous-intensity physical activity ≥ 3 days per week. Metabolic syndrome was defined when three or more of the following criteria were present in the collected data: triglyceride elevation (≥ 150 mg/dL) or use of related medication; decrease in high-density lipoprotein cholesterol (men: <40 mg/dL, women: <50 mg/dL) or use of related medication; blood pressure elevation (systolic ≥ 130 mmHg and/or diastolic ≥ 80 mmHg) or use of antihypertensive medications; fasting glucose elevation (≥ 100 mg/dL) or use of antidiabetic medications; and increased waist circumference (≥ 90 cm for Asian men, ≥ 80 cm for Asian women).

### Study outcomes

The primary outcome was the occurrence of MI and stroke. We investigated the association between age at smoking initiation, pack-years, and the risk of CCVDs. To minimize the cumulative effect of cigarette smoking due to an early age at smoking initiation, we analyzed the CCVDs risk and incidence rate based on the ratio of pack-years to the smoking initiation age.

### Statistical analysis

We utilized SAS software version 9.4 (SAS Institute Inc., Cary, NC, USA) to perform all statistical analyses. In the baseline characteristics, categorical variables were presented as numbers (percentages), and continuous variables were expressed as means (± standard deviation). We conducted Cox regression analysis to explore the potential association between the age at which individuals initiated smoking, smoking intensity, and the risk of CCVDs and mortality. Cox regression analysis was conducted to explore the independence of the associations after adjusting for potential confounding factors, including age, sex, income, alcohol consumption, physical activity, BMI, eGFR, metabolic syndrome, proteinuria, and pack-years. We validated the proportional hazards assumption using the log-log cumulative survival plot. The p-values were two-tailed, and the results were considered significant when the p-value was less than 0.05.

## Results

### Baseline characteristics

In total, 464,738 participants were included in this study. The baseline characteristics were compared according to smoking status, smoking initiation age, and pack-years (Table 1). The study groups were divided into four categories based on smoking initiation age and pack-years. The group with less than 20 pack-years and a smoking initiation age less than 20 years was defined as the smoking group 1. Smoking group 2 was designated as those with less than 20 pack-years and a smoking initiation age of 20 years or older, while smoking group 3 was assigned to individuals with 20 or more pack-years and a smoking initiation age of less than 20. Finally, the group with 20 or more pack-years and a smoking initiation age of 20 years or older was named the smoking group 4. The non-smoker, smoking group 1, smoking group 2, smoking group 3, and smoking group 4 comprised 364,513, 10,210, 42,601, 11,658, and 35,756 individuals, respective lt.

**Table 1:** Baseline characteristics of the study groups according to pack-year and smoking age

	Non smoker (*N* = 364,513)	Smoking group 1 (*N* = 10,210)	Smoking group 2 (*N* = 42,601)	Smoking group 3 (*N* = 11,658)	Smoking group 4 (*N* = 35,756)	*P*-value
**Age (yr)**	58.5 ± 14.69	35.03 ± 10.22	49.87 ± 13.01	53.14 ± 11.32	57.99 ± 10.41	< 0.001
**Sex (male)**	85,918(23.57)	9419(92.25)	35,186(82.59)	11,534(98.94)	34,265(95.83)	< 0.001
**Body shape measures**						
** Body mass index (kg/m**^**2**^)	24.19 ± 3.51	24.88 ± 4.33	24.54 ± 3.64	24.75 ± 3.52	24.33 ± 3.31	< 0.001
** Waist circumference (cm)**	80.7 ± 9.66	84.02 ± 10.63	84.07 ± 9.35	86.26 ± 8.72	85.54 ± 8.44	< 0.001
**Social and lifestyle factors**						
**Drinker** ^**a**^						< 0.001
Nondrinker	282,552(77.51)	2022(19.8)	13,888(32.6)	3421(29.34)	12,167(34.03)	
Mild drinker	75,362(20.67)	6084(59.59)	24,071(56.5)	4901(42.04)	16,787(46.95)	
Heavy drinker	6599(1.81)	2104(20.61)	4642(10.9)	3336(28.62)	6802(19.02)	
**Regular physical activity** ^**b**^	69,402(19.04)	1696(16.61)	8384(19.68)	1883(16.15)	6285(17.58)	< 0.001
**Low income** ^**c**^	92,685(25.43)	1780(17.43)	9662(22.68)	2781(23.85)	9173(25.65)	< 0.001
**Baseline comorbidities**						
** Diabetes mellitus**	74,045(20.31)	1120(10.97)	9682(22.73)	3787(32.48)	12,179(34.06)	< 0.001
** Hypertension**	190,948(52.38)	2580(25.27)	19,164(44.98)	6000(51.47)	20,567(57.52)	< 0.001
** Dyslipidemia**	129,979(35.66)	2032(19.9)	13,286(31.19)	4215(36.16)	13,444(37.6)	< 0.001
** Metabolic syndrome**	161,823(44.39)	2430(23.8)	16,859(39.57)	5573(47.8)	18,055(50.5)	< 0.001
**Laboratory measurements**						
** Systolic blood pressure (mmHg)**	125.96 ± 16.91	124.27 ± 15.66	126.45 ± 16.55	127.77 ± 16.52	128.45 ± 16.7	< 0.001
** Diastolic blood pressure (mmHg)**	77.17 ± 10.64	78.57 ± 11.31	79.19 ± 11.39	79.72 ± 11.26	79.48 ± 11.1	< 0.001
** Impaired fasting glucose**	104.28 ± 31.86	100.97 ± 33.56	109.6 ± 40.36	117.38 ± 46.95	116.93 ± 44.51	< 0.001
** Total cholesterol**	198.93 ± 40.42	195.8 ± 40.68	201.05 ± 41.58	200.51 ± 42.97	199.6 ± 42.18	< 0.001
** HDL**	55.5 ± 18.31	52.9 ± 17.73	52.3 ± 17.9	50.17 ± 17.98	50.19 ± 16.94	< 0.001
** LDL**	117.21 ± 36.76	111.31 ± 37.18	115.48 ± 39.52	113.51 ± 39.75	114.02 ± 39.25	< 0.001
** TG**	113.89(113.69-114.09)	136.96(135.27-138.66)	146.3(145.48-147.11)	163.67(161.97-165.39)	156.65(155.74-157.56)	< 0.001
** eGFR (mL/min/1.73 m**^**2**^)	64.53 ± 27.24	84.94 ± 37.34	73.09 ± 34.58	74.12 ± 34.57	70.5 ± 32.73	< 0.001
**Proteinuria**						< 0.001
Negative	230,105(63.13)	2139(20.95)	17,924(42.07)	4522(38.79)	16,677(46.64)	
1+	92,130(25.27)	5835(57.15)	16,956(39.8)	4755(40.79)	12,388(34.65)	
> 1+	42,278(11.6)	2236(21.9)	7721(18.12)	2381(20.42)	6691(18.71)	

Data are presented as the mean (1 standard deviation) for continuous variables or number (%) for categorical variables

HDL, high-density lipoprotein; LDL, low-density lipoprotein; TG, triglyceride; eGFR, estimated glomerular filtration rate

^a^There are three types of drinkers: nondrinker (0 g/day), mild drinker (0–30 g/day), and heavy drinker (≥ 30 g/day)

^b^Regular physical activity was defined as moderate-intensity physical activity ≥ 5 days or vigorous-intensity physical activity ≥ 3 days per week

^c^Individuals included in the lowest quartile (regarding required insurance fees or receiving free insurance) were categorized as the low-income group

Among the smoking groups, the largest number of participants (42,601 individuals) was in smoking group 2. This group started smoking after the age of 20 years and smoked for less than 20 pack-years. The age of participants in the smoking group varied across the subgroups. The smoking group 1 had the youngest average age at 35.03 ± 10.22, while smoking group 4 had the oldest average age at 57.99 ± 10.41. In non-smokers, the proportion of men was 23.57%, whereas in the smoking group, most groups had a higher prevalence of men. The proportion of heavy drinkers was higher in groups with a lower smoking initiation age. The rate of regular physical activity was lower in groups with a lower smoking initiation age. Regarding comorbid conditions such as diabetes, hypertension, hyperlipidemia, and metabolic syndrome, the smoking group showed an increasing prevalence of comorbidities with the age of the smokers. Group 4 had the highest incidence of comorbidities among patients with concurrent conditions.

### Risk of CCVDs and all-cause death according to the pack-year and smoking initiation age

The risk of CCVDs and all-cause death according to smoking initiation age and pack-years was also examined. Elevated risks of CCVDs and all-cause deaths were exhibited in smoking participants irrespective of smoking initiation age and pack-years, compared to the non-smoker group, even after multivariate analysis (Table 2).

**Table 2:** Risk of cardio-cerebrovascular and all-cause deaths according to pack-year and smoking age

	*N*	CCVDs	Duration	Incidence rate (/ 1000PY)	Univariate model	Multivariate model 1^a^	Multivariate model 2^b^	Death	Duration	Incidence rate (/ 1000PY)	Univariate model	Multivariate model 1^a^	Multivariate model 2^b^
Non smoker	364,513	31,421	3499724.46	8.98	1(Ref.)	1(Ref.)	1(Ref.)	38,495	3,288,132	11.7073	1(Ref.)	1(Ref.)	1(Ref.)
Pack-year < 20 & smoking age < 20	10,210	259	93268.15	2.78	0.32 (0.28,0.36)	1.26 (1.11,1.42)	1.27 (1.12,1.44)	329	94073.66	3.4973	0.3 (0.27,0.33)	2.12 (1.9,2.37)	1.93 (1.73,2.16)
Pack-year < 20 & smoking age ≥ 20	42,601	3164	371281.05	8.52	0.98 (0.95,1.02)	1.48 (1.42,1.54)	1.49 (1.43,1.55)	4212	381958.98	11.0274	0.95 (0.92,0.98)	1.66 (1.61,1.72)	1.6 (1.55,1.66)
Pack-year ≥ 20 & smoking age < 20	11,658	1274	97780.62	13.03	1.51 (1.43,1.6)	1.91 (1.8,2.03)	1.84 (1.73,1.95)	1721	102177.48	16.8432	1.45 (1.39,1.53)	2.08 (1.98,2.18)	1.9 (1.8,2)
Pack-year ≥ 20 & smoking age ≥ 20	35,756	4407	295153.84	14.93	1.73 (1.68,1.79)	1.68 (1.62,1.74)	1.66 (1.6,1.72)	6216	310687.91	20.0072	1.73 (1.68,1.77)	1.73 (1.68,1.78)	1.63 (1.58,1.68)
Pack-year < 30 & smoking age < 20	14,363	568	129478.5	4.387	0.5 (0.46,0.55)	1.53 (1.4,1.66)	1.53 (1.4,1.66)	721	131302.42	5.4911	0.47 (0.44,0.51)	2.26 (2.09,2.43)	2.08 (1.92,2.24)
Pack-year < 30 & smoking age ≥ 20	59,462	4927	513484.11	9.6	1.11 (1.07,1.14)	1.54 (1.48,1.59)	1.54 (1.49,1.59)	6578	530438.03	12.4011	1.07 (1.04,1.1)	1.67 (1.63,1.72)	1.61 (1.57,1.66)
Pack-year ≥ 30 & smoking age < 20	7505	965	61570.27	15.67	1.82 (1.7,1.94)	1.92 (1.8,2.05)	1.83 (1.71,1.96)	1329	64948.72	20.4623	1.77 (1.67,1.87)	2 (1.89,2.12)	1.82 (1.72,1.93)
Pack-year ≥ 30 & smoking age ≥ 20	18,895	2644	152950.78	17.29	2.01 (1.93,2.09)	1.7 (1.63,1.78)	1.66 (1.59,1.74)	3850	162208.87	23.7348	2.05 (1.98,2.12)	1.75 (1.69,1.81)	1.62 (1.57,1.69)
Pack-year < 40 & smoking age < 20	17,448	862	155637.52	5.54	0.64 (0.6,0.68)	1.6 (1.49,1.72)	1.59 (1.48,1.71)	1125	158471.23	7.0991	0.61 (0.58,0.65)	2.16 (2.03,2.3)	2 (1.88,2.13)
Pack-year < 40 & smoking age ≥ 20	70,012	6191	601367.73	10.3	1.19 (1.16,1.22)	1.56 (1.51,1.61)	1.56 (1.51,1.61)	8227	622853.38	13.2086	1.14 (1.11,1.16)	1.69 (1.64,1.73)	1.62 (1.57,1.66)
Pack-year ≥ 40 & smoking age < 20	4420	671	35411.26	18.95	2.2 (2.04,2.38)	1.99 (1.84,2.16)	1.88 (1.74,2.04)	925	37779.92	24.4839	2.12 (1.98,2.26)	2 (1.87,2.13)	1.79 (1.68,1.92)
Pack-year ≥ 40 & smoking age ≥ 20	8345	1380	65067.16	21.21	2.47 (2.34,2.61)	1.72 (1.62,1.82)	1.68 (1.59,1.78)	2201	69793.52	31.5359	2.73 (2.62,2.85)	1.76 (1.68,1.84)	1.62 (1.55,1.7)
Pack-year < 50 & smoking age < 20	19,334	1090	171420.02	6.36	0.73 (0.69,0.78)	1.66 (1.56,1.77)	1.63 (1.53,1.74)	1387	175114.31	7.9205	0.68 (0.65,0.72)	2.13 (2.02,2.25)	1.96 (1.85,2.08)
Pack-year < 50 & smoking age ≥ 20	75,300	7009	643718.96	10.89	1.26 (1.23,1.29)	1.58 (1.53,1.63)	1.57 (1.52,1.62)	9377	668,161	14.034	1.21 (1.18,1.24)	1.69 (1.64,1.73)	1.61 (1.57,1.65)
Pack-year ≥ 50 & smoking age < 20	2534	443	19628.76	22.57	2.63 (2.4,2.89)	2.02 (1.83,2.22)	1.91 (1.74,2.11)	663	21136.84	31.367	2.72 (2.52,2.94)	1.99 (1.84,2.15)	1.79 (1.65,1.94)
Pack-year ≥ 50 & smoking age ≥ 20	3057	562	22715.93	24.74	2.9 (2.67,3.15)	1.72 (1.57,1.87)	1.68 (1.54,1.83)	1051	24485.9	42.9227	3.75 (3.53,3.99)	1.83 (1.72,1.95)	1.68 (1.58,1.79)

^a^ Multivariate model 1 was adjusted for age, sex

^b^ Multivariate model 2 was adjusted for age, sex, alcohol consumption, regular physical activity, BMI, eGFR, proteinuria and metabolic syndrome

CCVDs, cardio-cerebrovascular diseases

**Fig. 2:**
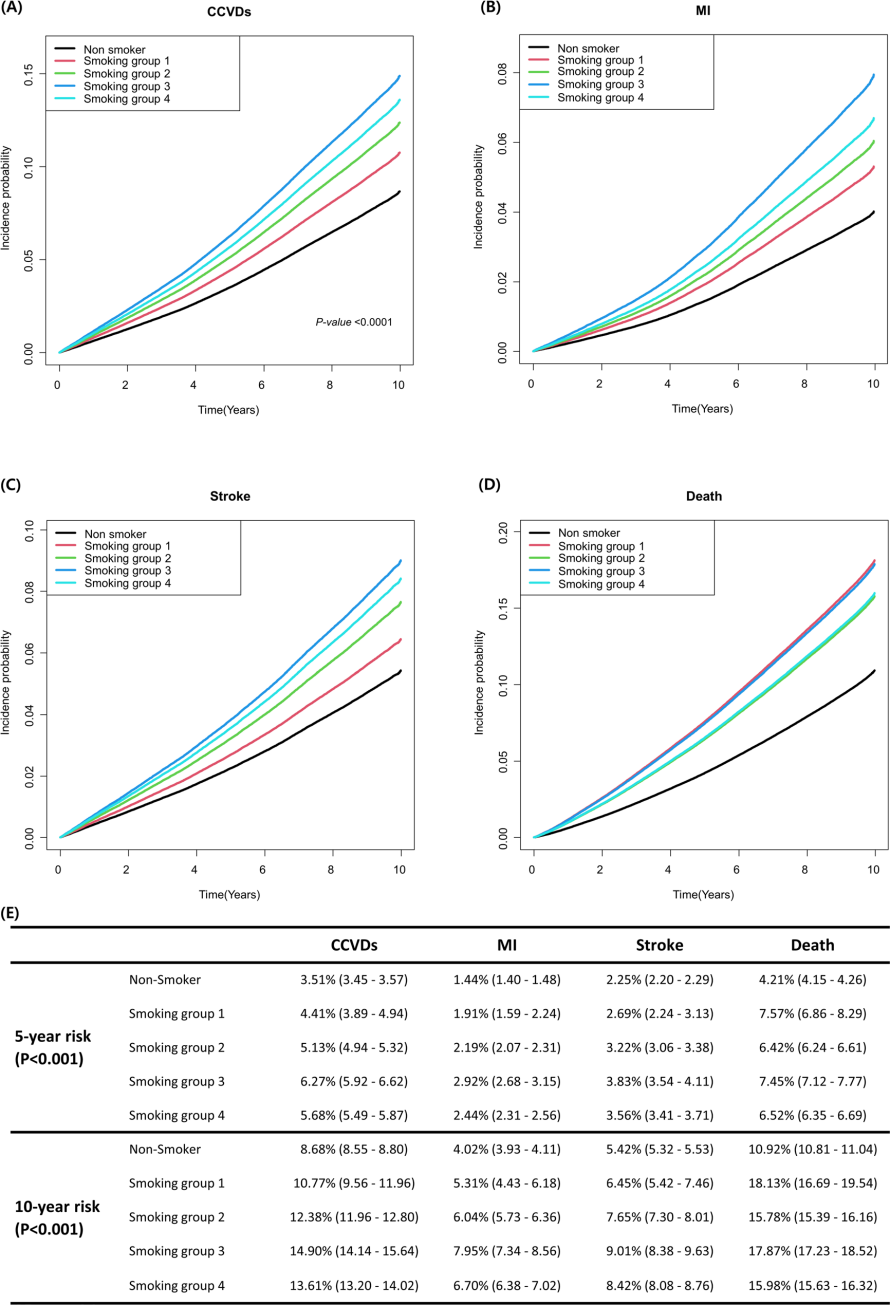
The risk of cardio-cerebrovascular and all-cause deaths according to smoking intensity and age at smoking initiation. In non-smoker and smoking groups, the highest risk for CCVDs, MI, and stroke was in patients in smoking group 3. Similarly, the 5- and 10-year risks of CCVDs, MI, and stroke were highest in smoking group 3. The risk of all-cause deaths was highest in smoking group 1, followed by smoking group 3. Smoking group 1, Pack-year < 20 &smoking age < 20; Smoking group 2, Pack-year < 20 &smoking age ≥ 20; Smoking group 3, Pack-year ≥ 20 &smoking age < 20; Smoking group 4, Pack-year ≥ 20 &smoking age ≥ 20; CCVDs, cardio-cerebrovascular diseases; MI, myocardial infarction

To compare the risk of CCVDs and all-cause death based on smoking intensity and initiation age, we categorized participants into four groups based on smoking intensity and initiation age and assessed the risk of adverse events. In the participants with pack years < 20, all-cause death was increased when the smoking initiation age was below 20 years (hazards ratio (HR) 1.93; 95% confidence interval (CI) 1.73–2.16). Since a lower smoking initiation age may have led to the increased pack-years, we conducted examinations in the high-risk groups, which had 30, 40, and 50 pack-years of smoking history. Participants who began smoking before the age of 20 showed an increased risk of all-cause mortality, regardless of smoking intensity. In this group, a higher pack-years was significantly associated with increased risks of CCVDs, MI, and stroke (Table 2 and Supplementary Table 1). Smoking group 3 (pack-years ≥ 20, smoking age < 20) was associated with the higher observed risks of CCVDs, MI, and stroke, while the highest risk of all-cause death was observed in smoking group 1 (Fig. 2).

**Fig. 3:**
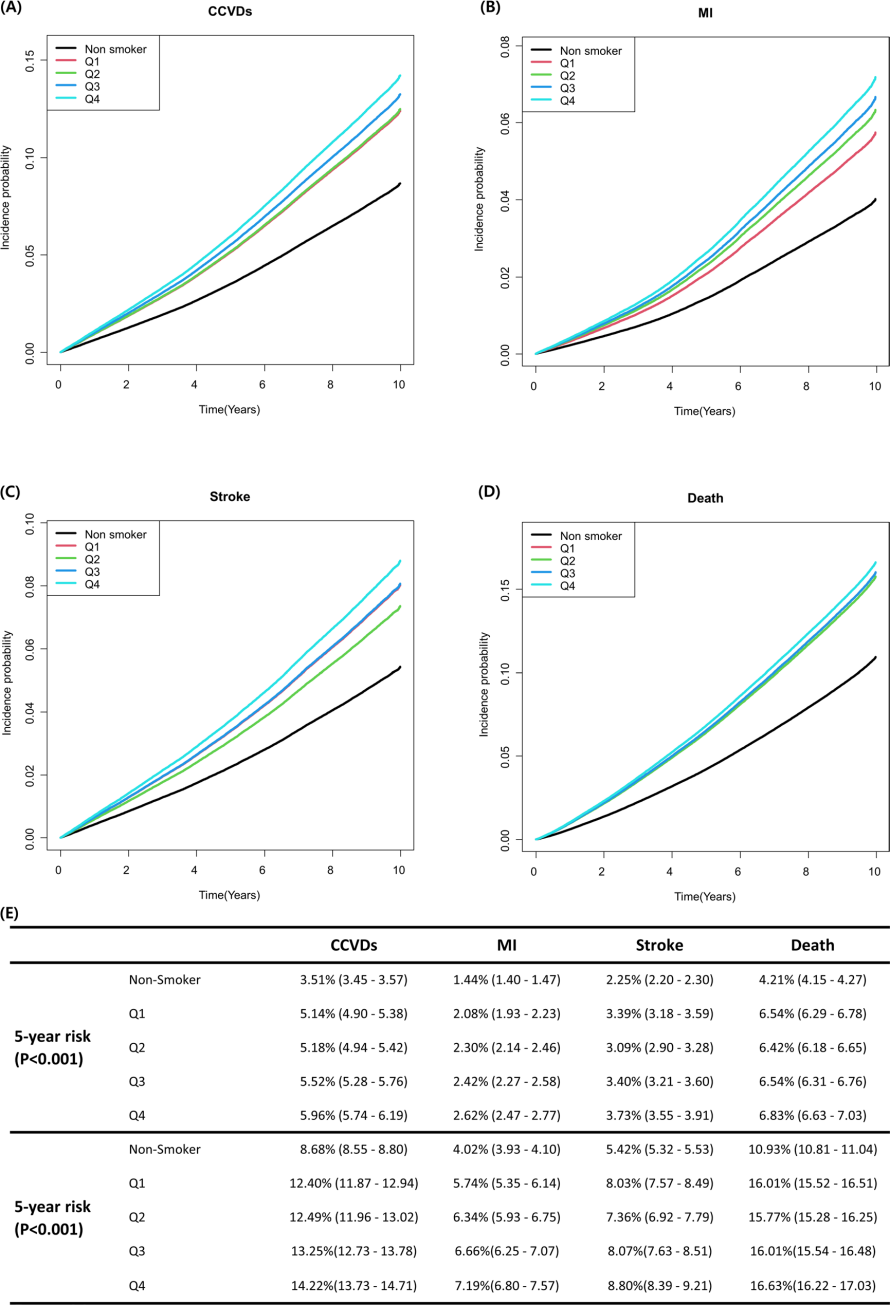
The risk of cardio-cerebrovascular and all-cause deaths by quartile of pack-year/age of smoking start. The group with the highest pack-year/smoking age ratio (Q4) had the highest rates of cardio-cerebrovascular and all-cause mortality. Similarly, the 5- and 10-year risks of CCVDs, MI, stroke and all-cause mortality were highest in the Q4 group

To mitigate the confounding bias related to the potential for higher smoking intensity with a younger smoking initiation age, we categorized the pack-year/smoking age ratio into quartiles (Table 3 and Supplementary Table 2). CCVDs and all-cause death were elevated in the group with a high pack-year/smoking age ratio (Q4), as depicted in Fig. 3, which illustrates the probability of CCVDs, MI, stroke, and all-cause death in a multivariate model based on pack-year/smoking age ratio quartiles.

**Table 3:** Risk by quartile of pack year/age of smoking start

Pack-years / Smoking age	*N*	CCVDs	Duration	Incidence rate (/ 1000PY)	Univariate model	Multivariate model 1^a^	Multivariate model2^b^	Death	Duration	Incidence rate (/ 1000PY)	Univariate model	Multivariate model 1^a^	Multivariate model2^b^
Non smoker	364,513	27,831	3196927.96	8.71	1(Ref.)	1(Ref.)	1(Ref.)	38,495	3,288,132	11.71	1(Ref.)	1(Ref.)	1(Ref.)
Q1	24,885	1752	217581.72	8.05	0.93 (0.88,0.97)	1.48 (1.41,1.56)	1.49 (1.42,1.57)	2411	223446.58	10.79	0.93 (0.89,0.97)	1.69 (1.62,1.76)	1.63 (1.56,1.7)
Q2	24,933	1895	216729.71	8.74	1.01 (0.96,1.06)	1.5 (1.42,1.57)	1.5 (1.43,1.58)	2567	223045.25	11.51	0.99 (0.95,1.03)	1.67 (1.6,1.74)	1.6 (1.53,1.67)
Q3	25,327	2248	217324.8	10.34	1.19 (1.14,1.25)	1.62 (1.55,1.7)	1.61 (1.53,1.68)	2926	225460.18	12.98	1.12 (1.08,1.16)	1.72 (1.65,1.79)	1.63 (1.57,1.7)
Q4	25,080	3209	205847.44	15.59	1.81 (1.74,1.88)	1.8 (1.72,1.87)	1.74 (1.67,1.82)	4574	216946.04	21.08	1.82 (1.77,1.88)	1.86 (1.8,1.93)	1.72 (1.66,1.78)
*P value*					< 0.001	< 0.001	< 0.001				< 0.001	< 0.001	< 0.001

^a^ Multivariate model 1 was adjusted for age, sex

^b^ Multivariate model 2 was adjusted for age, sex, alcohol consumption, regular physical activity, BMI, eGFR, proteinuria and metabolic syndrome

CCVDs, cardio-cerebrovascular diseases

Furthermore, to explore whether the risk of CCVDs, MI, stroke, and all-cause deaths increases with younger smoking initiation age, participants were reclassified based on their smoking initiation age. The risk factors according to age at smoking initiation were reanalyzed in multivariate model 3, which included pack-years as an adjustment factor, following multivariate model 2 (Table 4). As the age at smoking initiation decreased, the risks of CCVDs, MI, and stroke did not consistently increase. The highest risks of CCVDs, stroke and all-cause death were observed in the group that began smoking before the age of 15. Notably, the risk of all-cause deaths increased significantly with earlier smoking initiation, with the highest risk observed in those who started smoking before age 15 (HR 1.32; 95% CI 1.2–1.46).

**Table 4:** Comparison of the risk of deaths according to the age of smoking initiation

**Smoking age**	***N***	**CCVDs**	**Duration**	**Incidence rate (/1000PY)**	**Multivariate model2** ^**a**^	**Multivariate model3** ^**b**^	**MI**	**Duration**	**Incidence rate (/1000PY)**	**Multivariate model2** ^**a**^	**Multivariate model3** ^**b**^
< 15	2631	307	21601.62	14.21	1.3 (1.16,1.46)	1.1 (0.97,1.25)	131	22203.02	5.9	1.21 (1.01,1.446)	1 (0.83,1.21)
< 17	3694	297	31949.15	9.3	1.12 (1,1.27)	0.98 (0.86,1.11)	152	32473.03	4.68	1.22 (1.031,1.445)	1.05 (0.88,1.25)
< 20	15,543	929	137498.01	6.76	1.03 (0.96,1.12)	0.92 (0.85,1)	502	139037.35	3.61	1.15 (1.032,1.276)	1.01 (0.9,1.13)
< 25	31,358	2523	271892.67	9.28	1.05 (0.99,1.1)	0.96 (0.9,1.01)	1260	276682.48	4.55	1.1 (1.018,1.19)	1 (0.92,1.08)
< 30	16,630	1518	142038.8	10.69	1.02 (0.96,1.08)	0.96 (0.9,1.02)	715	145005.36	4.93	1.02 (0.936,1.121)	0.96 (0.87,1.05)
≥ 30	30,369	3530	252503.42	13.98	1(Ref.)	1(Ref.)	1604	259319.16	6.19	1(Ref.)	1(Ref.)
P for trend					0.0005	0.0649				0.0122	0.8924
**Smoking age**	**N**	**Stroke**	**Duration**	**Incidence rate (/1000PY)**	**Multivariate model2** ^**a**^	**Multivariate Model3** ^**b**^	**Death**	**Duration**	**Incidence rate (/1000PY)**	**Multivariate model2** ^**a**^	**Multivariate model3** ^**b**^
< 15	2631	206	21971.75	9.38	1.4 (1.21,1.62)	1.2 (1.03,1.4)	491	22634.39	21.69	1.37 (1.25,1.5)	1.32 (1.2,1.46)
< 17	3694	178	32347.72	5.5	1.11 (0.95,1.3)	0.97 (0.83,1.14)	423	32940.22	12.84	1.17 (1.06,1.3)	1.14 (1.03,1.27)
< 20	15,543	500	139004.2	3.6	0.97 (0.87,1.07)	0.87 (0.78,0.97)	1136	140676.54	8.08	1.12 (1.05,1.19)	1.09 (1.02,1.17)
< 25	31,358	1446	275620.89	5.25	1.01 (0.94,1.08)	0.93 (0.86,1.0)	3197	280798.85	11.39	1.03 (0.99,1.08)	1.01 (0.96,1.06)
< 30	16,630	913	144222.05	6.33	1.02 (0.94,1.1)	0.96 (0.88,1.04)	2062	147468.19	13.98	1.036 (0.98,1.09)	1.02 (0.97,1.08)
≥ 30	30,369	2198	257037.7	8.55	1(Ref.)	1(Ref.)	5169	264379.85	19.55	1(Ref.)	1(Ref.)
P for trend					0.0002	0.0017				< 0.001	< 0.001

^a^ Multivariate model 2 was adjusted for age, sex, alcohol consumption, regular physical activity, BMI, eGFR, proteinuria and metabolic syndrome

^b^ Multivariate model 3 was adjusted for age, sex, alcohol consumption, regular physical activity, BMI, eGFR, proteinuria, metabolic syndrome and pack-year

CCVDs, cardio-cerebrovascular diseases; MI, myocardial infarction

### Association of pack-year and risk of CCVDs, death, MI and stroke according to smoking age

The risks of CCVDs and mortality according to pack-years categorized according to smoking age were examined (Supplementary Table 3). When participants were stratified according to smoking initiation age, higher pack-years demonstrated a significant increase in the risk of CCVDs, MI, stroke, and all-cause death. Among the patients with CKD who started smoking before the age of 20, those with over 20 pack-years had an increased risk of CCVDs compared to those with less than 10 pack-years (HR 1.79; 95% CI 1.43–2.26). The risk of all-cause mortality according to pack-years was not statistically significant according to the age at which smoking began.

### Impact of smoking initiation age and intensity on CCVDs, MI, stroke, and mortality across demographics and clinical factors

The smoking intensity and smoking initiation age significantly impact the risk of CCVDs, MI, stroke, and all-cause mortality (Supplementary Table 4). These associations remained consistent across gender, age, alcohol consumption, physical activity, kidney function, and comorbidities (diabetes, hypertension, metabolic syndrome). Additionally, we presented stratified analyses in Supplementary Table 5, which demonstrated an increased risk in the smoking groups compared to non-smokers. These findings are consistent with our results of the risks of CCVDs, MI, stroke, and all-cause mortality according to pack-years and age at smoking initiation.

In summary, early smoking initiation and higher cumulative exposure estimated with pack-years were associated with elevated risks of CCVDs and all-cause mortality in patients with CKD. The risk was highest among those who started smoking before age 20 and those with accumulated over 20 pack-years.

## Discussion

In the present nationwide retrospective cohort study of patients with CKD, we investigated the associations between smoking initiation age, smoking intensity (pack-years), and the risks of CCVDs and all-cause mortality. The results indicated that both early smoking initiation and higher cumulative smoking exposure were independently associated with worse health outcomes in CKD patients. This study adds novel evidence detrimental effects of early smoking in individuals with CKD. The use of a comprehensive, population-based dataset with a large sample size strengthened the generalizability and reliability of the study results.

Excessive mortality among smokers is primarily caused by tobacco-related cancers, vascular diseases, and respiratory conditions [20, 21]. Numerous studies have shown that earlier smoking initiation is associated with higher risks of all-cause and cause-specific mortality in the general population [7–10]. In particular, individuals who began smoking before the age of 20 were found to have significantly increased risks of premature death compared to those who started after age 20 [7, 9]. A recent nationwide observational study further confirmed that both mortality risk and the benefits of smoking cessation are closely linked to the age of smoking initiation [10]. While the association between early smoking and mortality has been well established in the general population, limited evidence exists for patients with CKD. Our nationwide population-based cohort study addresses this gap and demonstrates that early smoking initiation is significantly associated with increased risks of CCVDs and all-cause mortality in individuals with CKD.

Prior research has consistently indicated that quitting smoking before the age of 30–40 significantly decreases mortality, highlighting the critical need for robust smoking cessation education and policies [7, 10]. Since childhood is a critical period for organ development, smoking during this time increases the risk of long-term morbidity and mortality [8]. Moreover, CKD independently elevates the risk of cardiovascular and cerebrovascular complications [11, 12]. Therefore, early smoking poses a particularly serious threat to individuals with CKD, highlighting the critical importance of early smoking cessation for mitigating health risks in the vulnerable population.

In this retrospective, nationwide cohort study from South Korea, we analyzed the impact of smoking intensity and age at smoking initiation on the risks of CCVDs and all-cause mortality in patients with CKD. These results suggest that early smoking initiation and higher smoking intensity are significantly associated with worse health outcomes in patients with CKD. Specifically, those who started smoking at a young age and had higher pack-years had an increased risk of CCVDs and all-cause mortality. Subgroup and stratified analyses further confirmed that these associations remained consistent across various demographic and clinical subgroups. These findings reinforce previous evidence linking early smoking to increased mortality and morbidity, and extend this knowledge to the high-risk CKD population.

Smoking is strongly associated with atherothrombotic vascular disease [22]. Several mechanisms may explain this association. Smoking induces a systemic inflammatory response, which plays a key role in the development and progression of atherosclerosis. Both male and female smokers have been shown to exhibit elevated levels of inflammatory markers, such as C-reactive protein, tumor necrosis factor-alpha, and interleukin-6 [23, 24]. Additionally, smoking contributes to insulin resistance and dyslipidemia, characterized by elevated triglycerides and reduced high-density lipoprotein levels, further elevating cardiovascular risk [25]. Based on previous findings, early exposure to smoking may exacerbate these pathological mechanisms and thereby worsen CKD-related complications.

Our findings suggest that early smoking initiation is an independent risk factor for CCVDs and all-cause mortality in CKD patients, even after adjusting for total smoking exposure (pack-years). Given the strong association between early smoking and adverse cardio-cerebrovascular outcomes, traditional CKD risk assessment models, which primarily focus on hypertension, diabetes, and proteinuria, may benefit from the inclusion of detailed smoking history.

From a clinical perspective, targeted interventions are warranted to mitigate the risks associated with early smoking in CKD patients. First, smoking history, including initiation age and cumulative exposure, should be routinely assessed during nephrology consultations. Second, personalized interventions should be developed for younger CKD patients with a history of early smoking, including intensified smoking cessation programs, close cardiovascular monitoring, and targeted education about the heightened risks of CCVDs. Future studies are needed to evaluate the effectiveness of integrating smoking history into CKD prediction models and assess whether targeted interventions based on smoking initiation age improve long-term patient outcomes. Enhancing CKD risk stratification through detailed smoking assessments may enable clinicians to deliver more individualized preventive strategies and improve prognosis in this high-risk population.

Considering the strong dose-response relationship between early smoking initiation and adverse outcomes observed in this study, targeted public health interventions are essential to reduce the long-term burden of CKD-related complications. School-based prevention programs and public education campaigns could enhance the prevention strategy. Moreover, comprehensive national smoking cessation campaigns could be strengthened for high-risk populations, including CKD patients with a history of early smoking.

This study has several strengths. It utilized a large, nationally representative cohort and adjusted for a wide range of potential confounders, including sociodemographic characteristics, lifestyle behaviors, and comorbidities. Moreover, the inclusion of pack-years as an adjustment variable allowed us to distinguish the effect of early smoking initiation from cumulative tobacco exposure.

However, some limitations must be acknowledged. First, the study population consisted predominantly of Koreans, which may limit the generalizability of the findings. Second, although we excluded patients who were already on dialysis or had received a kidney transplant, we could not determine the exact duration of CKD prior to study entry. Thus, the study cohort represents prevalent CKD cases rather than incident cases. This limitation should be considered when interpreting the generalizability of our findings. Third, as smoking history was self-reported during health examinations, recall bias may have affected the accuracy of smoking initiation age and pack-years. This could have led to misclassification of exposure levels and either underestimation or overestimation of the observed associations. Fourth, reverse causality may have led to underestimation of the risks or benefits of smoking and smoking cessation. Additionally, survival bias could not be ruled out, as individuals who began smoking at a very early age and experienced severe health consequences may have died before inclusion in the cohort. Finally, although we adjusted for multiple covariates, the possibility of residual or unmeasured confounding remains.

Future research is needed to explore the long-term effects of smoking cessation at different stages of CKD and evaluate whether early intervention can alter disease progression or cardiovascular outcomes. A better understanding of how smoking cessation impacts CKD-related risks could inform both clinical practice and public health strategies.

Taken together, these findings highlight the importance of considering both the age of smoking initiation and cumulative smoking exposure when assessing long-term prognosis in patients with CKD. Incorporating detailed smoking history into clinical risk assessments may improve the identification of high-risk individuals within this population.

## Conclusions

This observational study suggests that early smoking initiation is independently associated with elevated risks of both CCVDs and all-cause mortality in patients with CKD. These findings highlight the importance of preventing early smoking initiation and integrating smoking history into CKD risk assessment. Preventing early smoking initiation through strengthened public health policies and youth-targeted tobacco control strategies may contribute to reducing the long-term cardiovascular burden in this high-risk population. Future research should focus on refining risk prediction models and evaluating the effectiveness of tailored interventions for CKD patients with early smoking exposure.

## Electronic supplementary material

Below is the link to the electronic supplementary material.

Supplementary Material 1

## Data Availability

The data that support the findings of this study are available from NHID but restrictions apply to the availability of these data, which were used under license for the current study, and so are not publicly available. Data are however available from the authors upon reasonable request and with permission of NHID.

## Abbreviations

CKD: Chronic Kidney Disease.
CCVDs: Cardio-Cerebrovascular Diseases.
NHID: National Health Insurance Database.
NHIS: National Health Insurance Service.
CI: Confidence Interval.
IRB: Institutional Review Board.
