# Supplementary material for: Early smoking and its impact on cardio-cerebrovascular diseases in patients with chronic kidney disease: a nationwide population-based study
Source: BMC Public Health. 2025 Jun 3;25:2054. doi: 10.1186/s12889-025-23276-0 (PMC12131595; doi:10.1186/s12889-025-23276-0)
Supplement: Supplementary file 1 — Supplementary Material 1 [file 12889_2025_23276_MOESM1_ESM.docx]

**Supplementary table 1. Risk of myocardial infarction and stroke cause deaths according to pack-year and smoking age**

|  | N | MI | Duration | Incidence rate (/ 1000PY) | Univariate  model | Multivariate  model 1^a^ | Multivariate  model 2^b^ | Stroke | Duration | Incidence rate (/ 1000PY) | Univariate  model | Multivariate  model 1^a^ | Multivariate  model 2^b^ |
| --- | --- | --- | --- | --- | --- | --- | --- | --- | --- | --- | --- | --- | --- |
| Non smoker | 364513 | 12550 | 3249663.08 | 3.86194 | 1(Ref.) | 1(Ref.) | 1(Ref.) | 17270 | 3231302.8 | 5.34459 | 1(Ref.) | 1(Ref.) | 1(Ref.) |
| Pack-year < 20 &  smoking age < 20 | 10210 | 136 | 93667.14 | 1.45195 | 0.38  (0.32,0.45) | 1.26  (1.06,1.5) | 1.34  (1.12,1.59) | 138 | 93641.55 | 1.4737 | 0.28  (0.23,0.33) | 1.23  (1.04,1.46) | 1.2  (1.01,1.43) |
| Pack-year < 20 &  smoking age ≥ 20 | 42601 | 1516 | 377064.21 | 4.02054 | 1.05  (0.99,1.1) | 1.48  (1.4,1.57) | 1.53  (1.45,1.63) | 1869 | 375703.63 | 4.97467 | 0.93  (0.89,0.98) | 1.47  (1.4,1.55) | 1.45  (1.37,1.52) |
| Pack-year ≥ 20 &  smoking age < 20 | 11658 | 649 | 100046.27 | 6.487 | 1.7  (1.57,1.84) | 2.03  (1.87,2.21) | 2.06  (1.89,2.24) | 746 | 99682.13 | 7.48379 | 1.41  (1.31,1.52) | 1.86  (1.72,2.01) | 1.73  (1.6,1.87) |
| Pack-year ≥ 20 &  smoking age ≥ 20 | 35756 | 2063 | 303942.79 | 6.78746 | 1.78  (1.7,1.86) | 1.67  (1.59,1.76) | 1.71  (1.62,1.81) | 2688 | 301177.01 | 8.92498 | 1.68  (1.61,1.75) | 1.68  (1.61,1.76) | 1.61  (1.53,1.68) |
| Pack-year < 30 &  smoking age < 20 | 14363 | 299 | 130375.79 | 2.29337 | 0.59  (0.53,0.67) | 1.58  (1.4,1.78) | 1.66  (1.47,1.87) | 302 | 130334.4 | 2.3171 | 0.43  (0.39,0.49) | 1.45  (1.29,1.64) | 1.41  (1.25,1.58) |
| Pack-year < 30 &  smoking age ≥ 20 | 59462 | 2349 | 522729.64 | 4.49372 | 1.17  (1.12,1.22) | 1.54  (1.46,1.61) | 1.59  (1.51,1.67) | 2917 | 520466.82 | 5.6046 | 1.05  (1.01,1.1) | 1.52  (1.46,1.59) | 1.49  (1.43,1.56) |
| Pack-year ≥ 30 &  smoking age < 20 | 7505 | 486 | 63337.62 | 7.67316 | 2.01  (1.84,2.2) | 2.04  (1.86,2.24) | 2.05  (1.86,2.25) | 582 | 62989.28 | 9.2397 | 1.74  (1.6,1.89) | 1.91  (1.75,2.08) | 1.76  (1.61,1.92) |
| Pack-year ≥ 30 &  smoking age ≥ 20 | 18895 | 1230 | 158277.36 | 7.77117 | 2.04  (1.92,2.16) | 1.69  (1.59,1.8) | 1.72  (1.61,1.83) | 1640 | 156413.82 | 10.485 | 1.98  (1.88,2.08) | 1.72  (1.63,1.82) | 1.62  (1.53,1.72) |
| Pack-year < 40 &  smoking age < 20 | 17448 | 459 | 157014.32 | 2.9233 | 0.76  (0.69,0.83) | 1.71  (1.55,1.89) | 1.78  (1.61,1.97) | 463 | 156971.09 | 2.9496 | 0.55  (0.5,0.61) | 1.51  (1.37,1.66) | 1.45  (1.314,1.595) |
| Pack-year < 40 &  smoking age ≥ 20 | 70012 | 2969 | 613046.9 | 4.84302 | 1.26  (1.21,1.31) | 1.58  (1.5,1.65) | 1.62  (1.55,1.7) | 3652 | 610230.44 | 5.9846 | 1.12  (1.09,1.17) | 1.54  (1.48,1.61) | 1.5  (1.43,1.56) |
| Pack-year ≥ 40 &  smoking age < 20 | 4420 | 326 | 36699.09 | 8.88305 | 2.33  (2.09,2.6) | 2.05  (1.83,2.3) | 2.03  (1.81,2.28) | 421 | 36352.59 | 11.581 | 2.18  (1.98,2.41) | 2.04  (1.84,2.25) | 1.85  (1.68,2.05) |
| Pack-year ≥ 40 &  smoking age ≥ 20 | 8345 | 610 | 67960.1 | 8.97585 | 2.36  (2.18,2.56) | 1.63  (1.5,1.78) | 1.67  (1.53,1.82) | 905 | 66650.2 | 13.5784 | 2.57  (2.4,2.74) | 1.81  (1.68,1.94) | 1.71  (1.6,1.84) |
| Pack-year < 50 &  smoking age < 20 | 19334 | 580 | 173192.83 | 3.34887 | 0.87  (0.8,0.95) | 1.791  (1.64,1.96) | 1.85  (1.69,2.02) | 585 | 173185.94 | 3.3779 | 0.63  (0.58,0.69) | 1.55  (1.42,1.69) | 1.47  (1.34,1.6) |
| Pack-year < 50 &  smoking age ≥ 20 | 75300 | 3343 | 657201.24 | 5.08672 | 1.33  (1.28,1.38) | 1.59  (1.52,1.66) | 1.63  (1.56,1.71) | 4179 | 653580.08 | 6.394 | 1.2  (1.16,1.24) | 1.57  (1.51,1.63) | 1.52  (1.46,1.58) |
| Pack-year ≥ 50 &  smoking age < 20 | 2534 | 205 | 20520.58 | 9.98997 | 2.63  (2.3,3.02) | 2  (1.74,2.3) | 1.98  (1.72,2.28) | 299 | 20137.73 | 14.8477 | 2.81  (2.51,3.15) | 2.19  (1.95,2.46) | 2.01  (1.78,2.26) |
| Pack-year ≥ 50 &  smoking age ≥ 20 | 3057 | 236 | 23805.76 | 9.91357 | 2.63  (2.31,2.99) | 1.58  (1.38,1.8) | 1.61  (1.41,1.83) | 378 | 23300.56 | 16.2228 | 3.08  (2.78,3.41) | 1.81  (1.63,2.01) | 1.72  (1.55,1.91) |

^a^ Multivariate model 1 was adjusted for age, sex.

^b^ Multivariate model 2 was adjusted for age, sex, alcohol consumption, regular physical activity, BMI, eGFR, proteinuria and metabolic syndrome.

MI, myocardial infarction

**Supplementary table 2. Risk of myocardial infarction and stroke cause deaths by quartile of pack year/age of smoking start**

| Pack-years / smoking age | N | MI | Duration | Incidence rate (/ 1000PY) | Univariate  model | Multivariate  model 1^a^ | Multivariate  model 2^b^ | Stroke | Duration | Incidence rate (/ 1000PY) | Univariate  model | Multivariate  model 1^a^ | Multivariate  model 2^b^ |
| --- | --- | --- | --- | --- | --- | --- | --- | --- | --- | --- | --- | --- | --- |
| Non smoker | 364513 | 12550 | 3249663.08 | 3.86 | 1(Ref.) | 1(Ref.) | 1(Ref.) | 17270 | 3231302.8 | 5.34 | 1(Ref.) | 1(Ref.) | 1(Ref.) |
| Q1 | 24885 | 794 | 220885.93 | 3.59 | 0.94  (0.87,1) | 1.41  (1.31,1.52) | 1.45  (1.35,1.57) | 1092 | 219881.36 | 4.97 | 0.93  (0.88,0.99) | 1.55  (1.45,1.65) | 1.53  (1.43,1.63) |
| Q2 | 24933 | 939 | 220062.38 | 4.27 | 1.11  (1.04,1.19) | 1.56  (1.45,1.67) | 1.61  (1.5,1.73) | 1070 | 219454.18 | 4.88 | 0.92  (0.86,0.97) | 1.42  (1.33,1.51) | 1.39  (1.3,1.48) |
| Q3 | 25327 | 1099 | 221684.38 | 4.96 | 1.29  (1.21,1.37) | 1.66  (1.55,1.77) | 1.7  (1.59,1.82) | 1317 | 220716.96 | 5.97 | 1.12  (1.06,1.19) | 1.59  (1.5,1.69) | 1.53  (1.44,1.63) |
| Q4 | 25080 | 1532 | 212087.73 | 7.22 | 1.89  (1.79,1.99) | 1.82  (1.71,1.93) | 1.85  (1.74,1.96) | 1962 | 210151.81 | 9.34 | 1.76  (1.68,1.84) | 1.8  (1.71,1.89) | 1.69  (1.6,1.78) |
| *P value* |  |  |  |  | <0.001 | <0.001 | <0.001 |  |  |  | <0.001 | <0.001 | <0.001 |

^a^ Multivariate model 1 was adjusted for age, sex.

^b^ Multivariate model 2 was adjusted for age, sex, income status, alcohol consumption, regular physical activity, BMI, eGFR, proteinuria and metabolic syndrome.

MI, myocardial infarction

**Supplementary Table 3. The risk of cardio-cerebrovascular diseases, death, myocardial infarction and stroke according to pack years, categorized by smoking initiation age**

| Smoking age | Pack-years | N | CCVDs | Duration | IR (per 1,000) | Model 2^a^ | *P value* | Death | Duration | IR (per 1,000) | Model 2^a^ | *P value* |
| --- | --- | --- | --- | --- | --- | --- | --- | --- | --- | --- | --- | --- |
| < 20 | < 10 | 4762 | 80 | 43916.96 | 1.82 | 1(Ref.) | <0.001 | 95 | 44130.78 | 2.16 | 1(Ref.) | 0.699 |
|  | < 15 | 2803 | 70 | 25634.3 | 2.73 | 0.99  (0.72,1.37) |  | 87 | 25839.98 | 3.37 | 0.83  (0.62,1.11) |  |
|  | < 20 | 2645 | 109 | 23716.9 | 4.6 | 1.26  (0.94,1.68) |  | 147 | 24102.9 | 6.1 | 0.9  (0.69,1.16) |  |
|  | ≥ 20 | 11658 | 1274 | 97780.62 | 13.03 | 1.79  (1.43,2.26) |  | 1721 | 102177.48 | 16.84 | 0.97  (0.79,1.19) |  |
| < 25 | < 10 | 5448 | 137 | 49903.04 | 2.75 | 1(Ref.) |  | 165 | 50316.26 | 3.28 | 1(Ref.) |  |
|  | < 15 | 4096 | 196 | 36690.8 | 5.34 | 1.31  (1.05,1.63) |  | 221 | 37332.14 | 5.92 | 0.97  (0.79,1.18) |  |
|  | < 20 | 3861 | 225 | 34188.9 | 6.58 | 1.27  (1.03,1.58) |  | 257 | 35039.9 | 7.33 | 0.85  (0.7,1.04) |  |
|  | ≥ 20 | 17953 | 1965 | 151109.93 | 13 | 1.58  (1.32,1.88) |  | 2554 | 158110.54 | 16.15 | 0.96  (0.82,1.12) |  |
| < 30 | < 10 | 3340 | 121 | 30093.36 | 4.02 | 1(Ref.) |  | 166 | 30497.48 | 5.44 | 1(Ref.) |  |
|  | < 15 | 2251 | 142 | 19772.56 | 7.18 | 1.16  (0.91,1.48) |  | 197 | 20271.45 | 9.72 | 0.96  (0.78,1.18) |  |
|  | < 20 | 2413 | 174 | 20994 | 8.29 | 1.14  (0.91,1.44) |  | 221 | 21664.75 | 10.2 | 0.86  (0.7,1.05) |  |
|  | ≥ 20 | 8626 | 1081 | 71178.88 | 15.19 | 1.45  (1.19,1.75) |  | 1478 | 75034.51 | 19.7 | 0.91  (0.78,1.07) |  |
| ≥ 30 | < 10 | 12398 | 1089 | 106457.19 | 10.23 | 1(Ref.) |  | 1532 | 110051.52 | 13.92 | 1(Ref.) |  |
|  | < 15 | 5066 | 581 | 42441.41 | 13.69 | 1.1  (0.99,1.22) |  | 786 | 44448.09 | 17.68 | 0.95  (0.87,1.03) |  |
|  | < 20 | 3728 | 499 | 30739.79 | 16.23 | 1.09  (0.98,1.22) |  | 667 | 32337.39 | 20.63 | 0.88  (0.8,0.96) |  |
|  | ≥ 20 | 9177 | 1361 | 72865.04 | 18.68 | 1.15  (1.06,1.25) |  | 2184 | 77542.86 | 28.17 | 1.03  (0.96,1.1) |  |
| Smoking age | Pack-years | N | MI | Duration | IR (per 1,000) | Model 2^a^ | *P value* | Stroke | Duration | IR (per 1,000) | Model 2^a^ | *P value* |
| < 20 | < 10 | 4762 | 50 | 44001.02 | 1.14 | 1(Ref.) | 0.0005 | 34 | 44037.05 | 0.77 | 1(Ref.) | 0.0055 |
|  | < 15 | 2803 | 36 | 25724.65 | 1.4 | 0.89  (0.58,1.36) |  | 40 | 25738.76 | 1.55 | 1.23  (0.78,1.94) |  |
|  | < 20 | 2645 | 50 | 23941.47 | 2.09 | 1.05  (0.71,1.56) |  | 64 | 23865.73 | 2.68 | 1.49  (0.98,2.27) |  |
|  | ≥ 20 | 11658 | 649 | 100046.27 | 6.49 | 1.86  (1.38,2.5) |  | 746 | 99682.13 | 7.48 | 1.9  (1.34,2.69) |  |
| < 25 | < 10 | 5448 | 74 | 50094.08 | 1.48 | 1(Ref.) |  | 69 | 50116.93 | 1.38 | 1(Ref.) |  |
|  | < 15 | 4096 | 109 | 37000.41 | 2.95 | 1.44  (1.07,1.94) |  | 97 | 37009.16 | 2.62 | 1.2  (0.88,1.63) |  |
|  | < 20 | 3861 | 133 | 34595.55 | 3.84 | 1.55  (1.16,2.06) |  | 112 | 34582.76 | 3.24 | 1.13  (0.83,1.52) |  |
|  | ≥ 20 | 17953 | 944 | 154992.44 | 6.09 | 1.68  (1.32,2.14) |  | 1168 | 153912.02 | 7.59 | 1.53  (1.2,1.96) |  |
| < 30 | < 10 | 3340 | 52 | 30342.07 | 1.71 | 1(Ref.) |  | 74 | 30237.76 | 2.45 | 1(Ref.) |  |
|  | < 15 | 2251 | 71 | 20035.85 | 3.54 | 1.45  (1.01,2.07) |  | 80 | 19971.47 | 4.01 | 1.0  (0.73,1.37) |  |
|  | < 20 | 2413 | 91 | 21305.81 | 4.27 | 1.53  (1.09,2.15) |  | 94 | 21321.65 | 4.41 | 0.92  (0.68,1.25) |  |
|  | ≥ 20 | 8626 | 501 | 73321.62 | 6.83 | 1.81  (1.36,2.42) |  | 665 | 72691.17 | 9.15 | 1.23  (0.97,1.57) |  |
| ≥ 30 | < 10 | 12398 | 483 | 108519.05 | 4.45 | 1(Ref.) |  | 697 | 107805.52 | 6.47 | 1(Ref.) |  |
|  | < 15 | 5066 | 270 | 43549.32 | 6.2 | 1.18  (1.02,1.37) |  | 358 | 43247.02 | 8.28 | 1.02  (0.9,1.16) |  |
|  | < 20 | 3728 | 233 | 31622.07 | 7.37 | 1.21  (1.03,1.42) |  | 288 | 31411.36 | 9.17 | 0.94  (0.82,1.08) |  |
|  | ≥ 20 | 9177 | 618 | 75628.72 | 8.17 | 1.26  (1.11,1.42) |  | 855 | 74573.81 | 11.47 | 1.04  (0.94,1.16) |  |

^a^ Multivariate model 2 was adjusted for age, sex, income status, alcohol consumption, regular physical activity, BMI, eGFR, proteinuria and metabolic syndrome.

CCVDs, cardio-cerebrovascular diseases; MI, myocardial infarction; IR, incidence rate

**Supplementary Table 4. Adjusted risk of cardio-cerebrovascular diseases, death, myocardial infarction and stroke by smoking exposure and related risk factors.**

|  |  | N | CCVDs | Duration | IR (per 1,000) | Model 2^a^ | *P* for interaction | MI | Duration | IR (per 1,000) | Model 2^a^ | *P* for interaction | Stroke | Duration | IR (per 1,000) | Model 2^a^ | *P* for interaction | Death | Duration | IR (per 1,000) | Model 2^a^ | *P* for interaction |
| --- | --- | --- | --- | --- | --- | --- | --- | --- | --- | --- | --- | --- | --- | --- | --- | --- | --- | --- | --- | --- | --- | --- |
| Age < 65 | Non smoker | 224690 | 8527 | 2052247.68 | 4.16 | 1(Ref.) | <.0001 | 4196 | 2068400.13 | 2.03 | 1(Ref.) | <.0001 | 4806 | 2065179.7 | 2.3272 | 1(Ref.) | <.0001 | 6866 | 2082333.33 | 3.3 | 1(Ref.) | <.0001 |
|  | Smoking group 1 | 9923 | 199 | 91341.78 | 2.18 | 1.22  (1.06,1.41) |  | 112 | 91644.95 | 1.22 | 1.42  (1.17,1.72) |  | 98 | 91647.8 | 1.0693 | 1.08  (0.88,1.32) |  | 196 | 91977.64 | 2.13 | 2.5  (2.16,2.88) |  |
|  | Smoking group 2 | 36380 | 2017 | 325152.44 | 6.2 | 1.59  (1.51,1.68) |  | 1047 | 328766.19 | 3.18 | 1.72  (1.6,1.85) |  | 1080 | 328564.88 | 3.287 | 1.48  (1.38,1.58) |  | 1890 | 332448.92 | 5.69 | 1.86  (1.77,1.96) |  |
|  | Smoking group 3 | 9600 | 846 | 83316.54 | 10.15 | 2.03  (1.89,2.19) |  | 442 | 84842.8 | 5.21 | 2.25  (2.03,2.5) |  | 465 | 84801.94 | 5.4834 | 1.87  (1.69,2.07) |  | 853 | 86475.28 | 9.86 | 2.24  (2.08,2.41) |  |
|  | Smoking group 4 | 26178 | 2597 | 225365.58 | 11.524 | 1.91  (1.82,2) |  | 1319 | 230565.33 | 5.72 | 2.05  (1.91,2.19) |  | 1472 | 229481.47 | 6.4145 | 1.82  (1.7,1.94) |  | 2557 | 235113.22 | 10.88 | 1.9  (1.81,2) |  |
| Age ≥ 65 | Non smoker | 139823 | 19304 | 1144680.28 | 16.86 | 1(Ref.) |  | 8354 | 1181262.95 | 7.07 | 1(Ref.) |  | 12464 | 1166123.1 | 10.6884 | 1(Ref.) |  | 31629 | 1205798.68 | 26.23 | 1(Ref.) |  |
|  | Smoking group 1 | 287 | 60 | 1926.37 | 31.15 | 1.56  (1.21,2.01) |  | 24 | 2022.18 | 11.87 | 1.45  (0.97,2.16) |  | 40 | 1993.75 | 20.0627 | 1.55  (1.13,2.11) |  | 133 | 2096.02 | 63.45 | 1.56  (1.32,1.85) |  |
|  | Smoking group 2 | 6221 | 1147 | 46128.61 | 24.87 | 1.39  (1.31,1.48) |  | 469 | 48298.02 | 9.71 | 1.33  (1.21,1.46) |  | 789 | 47138.75 | 16.7378 | 1.45  (1.34,1.56) |  | 2322 | 49510.06 | 46.9 | 1.48  (1.42,1.55) |  |
|  | Smoking group 3 | 2058 | 428 | 14464.08 | 29.59 | 1.64  (1.49,1.81) |  | 207 | 15203.47 | 13.62 | 1.87  (1.63,2.15) |  | 281 | 14880.18 | 18.8842 | 1.6  (1.42,1.81) |  | 868 | 15702.2 | 55.28 | 1.72  (1.6,1.84) |  |
|  | Smoking group 4 | 9578 | 1810 | 69788.26 | 25.94 | 1.44  (1.37,1.514) |  | 744 | 73377.46 | 10.14 | 1.38  (1.27,1.49) |  | 1216 | 71695.54 | 16.9606 | 1.45  (1.36,1.54) |  | 3659 | 75574.69 | 48.42 | 1.52  (1.46,1.58) |  |
| Male | Non smoker | 85918 | 7748 | 732526.46 | 10.58 | 1(Ref.) | <.0001 | 3526 | 746560.88 | 4.72 | 1(Ref.) | 0.0015 | 4807 | 741777.92 | 6.48 | 1(Ref.) | <.0001 | 12530 | 756913.92 | 16.55 | 1(Ref.) | <.0001 |
|  | Smoking group 1 | 9419 | 239 | 86000.73 | 2.78 | 1.19  (1.05,1.36) |  | 124 | 86388.03 | 1.44 | 1.24  (1.04,1.49) |  | 129 | 86341.2 | 1.49 | 1.142  (0.96,1.36) |  | 307 | 86761.57 | 3.54 | 1.85  (1.65,2.07) |  |
|  | Smoking group 2 | 35186 | 2505 | 306777.64 | 8.17 | 1.41  (1.34,1.47) |  | 1221 | 311293.91 | 3.92 | 1.47  (1.38,1.57) |  | 1450 | 310333.09 | 4.67 | 1.35  (1.27,1.43) |  | 3432 | 315212.16 | 10.89 | 1.57  (1.51,1.63) |  |
|  | Smoking group 3 | 11534 | 1259 | 96742.27 | 13.01 | 1.81  (1.7,1.92) |  | 642 | 98976.57 | 6.49 | 2.03  (1.86,2.21) |  | 737 | 98631.4 | 7.47 | 1.7  (1.57,1.84) |  | 1692 | 101095.19 | 16.74 | 1.87  (1.78,1.97) |  |
|  | Smoking group 4 | 34265 | 4184 | 283176.59 | 14.78 | 1.62  (1.56,1.68) |  | 1957 | 291542.47 | 6.71 | 1.68  (1.58,1.77) |  | 2555 | 288871.67 | 8.84 | 1.57  (1.5,1.65) |  | 5869 | 297925.96 | 19.7 | 1.6  (1.55,1.65) |  |
| Female | Non smoker | 278595 | 20083 | 2464401.5 | 8.15 | 1(Ref.) |  | 9024 | 2503102.21 | 3.61 | 1(Ref.) |  | 12463 | 2489524.88 | 5.01 | 1(Ref.) |  | 25965 | 2531218.08 | 10.26 | 1(Ref.) |  |
|  | Smoking group 1 | 791 | 20 | 7267.43 | 2.75 | 2.74  (1.77,4.25) |  | 12 | 7279.11 | 1.65 | 3.3  (1.87,5.82) |  | 9 | 7300.35 | 1.23 | 2.14  (1.11,4.12) |  | 22 | 7312.1 | 3.01 | 3.81  (2.51,5.79) |  |
|  | Smoking group 2 | 7415 | 659 | 64503.41 | 10.22 | 1.76  (1.63,1.9) |  | 295 | 65770.3 | 4.49 | 1.73  (1.54,1.94) |  | 419 | 65370.54 | 6.41 | 1.79  (1.63,1.98) |  | 780 | 66746.83 | 11.69 | 1.67  (1.56,1.8) |  |
|  | Smoking group 3 | 124 | 15 | 1038.35 | 14.45 | 1.65  (1,2.74) |  | 7 | 1069.7 | 6.54 | 1.75  (0.83,3.66) |  | 9 | 1050.73 | 8.57 | 1.58  (0.82,3.03) |  | 29 | 1082.29 | 26.8 | 2.18  (1.51,3.13) |  |
|  | Smoking group 4 | 1491 | 223 | 11977.25 | 18.62 | 1.81  (1.59,2.07) |  | 106 | 12400.32 | 8.55 | 1.95  (1.61,2.36) |  | 133 | 12305.34 | 10.81 | 1.66  (1.4,1.98) |  | 347 | 12761.95 | 27.19 | 1.96  (1.76,2.18) |  |
| BMI < 25 | Non smoker | 224070 | 15633 | 1963391.19 | 7.96 | 1(Ref.) | 0.0279 | 7023 | 1992328.61 | 3.53 | 1(Ref.) | 0.2781 | 9731 | 1981860.4 | 4.91 | 1(Ref.) | 0.0027 | 24669 | 2013068.32 | 12.25 | 1(Ref.) | <.0001 |
|  | Smoking group 1 | 5593 | 147 | 50953.22 | 2.89 | 1.32  (1.12,1.56) |  | 77 | 51145.33 | 1.51 | 1.41  (1.13,1.77) |  | 80 | 51164.87 | 1.56 | 1.25  (1,1.56) |  | 222 | 51373.32 | 4.32 | 1.86  (1.63,2.13) |  |
|  | Smoking group 2 | 24247 | 1876 | 208780.88 | 8.99 | 1.5  (1.43,1.6) |  | 861 | 212208.19 | 4.06 | 1.5  (1.39,1.62) |  | 1155 | 211063.33 | 5.47 | 1.51  (1.41,1.61) |  | 3009 | 214783.36 | 14.01 | 1.65  (1.59,1.72) |  |
|  | Smoking group 3 | 6423 | 747 | 52448.87 | 14.24 | 1.85  (1.71,2) |  | 360 | 53833.87 | 6.69 | 1.98  (1.77,2.21) |  | 465 | 53408.42 | 8.71 | 1.83  (1.66,2.01) |  | 1248 | 54940.19 | 22.72 | 1.99  (1.88,2.11) |  |
|  | Smoking group 4 | 21221 | 2794 | 171269.59 | 16.31 | 1.73  (1.65,1.8) |  | 1277 | 176782.72 | 7.22 | 1.76  (1.65,1.88) |  | 1746 | 174778.13 | 9.99 | 1.7  (1.6,1.8) |  | 4516 | 180762.98 | 24.98 | 1.68  (1.62,1.74) |  |
| BMI ≥ 25 | Non smoker | 140443 | 12198 | 1233536.77 | 9.89 | 1(Ref.) |  | 5527 | 1257334.47 | 4.4 | 1(Ref.) |  | 7539 | 1249442.4 | 6.03 | 1(Ref.) |  | 13826 | 1275063.69 | 10.84 | 1(Ref.) |  |
|  | Smoking group 1 | 4617 | 112 | 42314.93 | 2.65 | 1.2  (1,1.45) |  | 59 | 42521.81 | 1.39 | 1.25  (0.96,1.62) |  | 58 | 42476.68 | 1.37 | 1.13  (0.87,1.47) |  | 107 | 42700.35 | 2.51 | 2.09  (1.73,2.53) |  |
|  | Smoking group 2 | 18354 | 1288 | 162500.17 | 7.93 | 1.46  (1.38,1.56) |  | 655 | 164856.03 | 3.97 | 1.58  (1.45,1.72) |  | 714 | 164640.31 | 4.34 | 1.36  (1.26,1.48) |  | 1203 | 167175.62 | 7.2 | 1.48(1.39,1.57) |  |
|  | Smoking group 3 | 5235 | 527 | 45331.75 | 11.63 | 1.82  (1.67,1.99) |  | 289 | 46212.4 | 6.25 | 2.16  (1.91,2.44) |  | 281 | 46273.7 | 6.07 | 1.59  (1.41,1.8) |  | 473 | 47237.29 | 10.01 | 1.69  (1.54,1.86) |  |
|  | Smoking group 4 | 14535 | 1613 | 123884.25 | 13.02 | 1.55  (1.47,1.64) |  | 786 | 127160.07 | 6.18 | 1.64  (1.51,1.78) |  | 942 | 126398.87 | 7.45 | 1.47  (1.36,1.58) |  | 1700 | 129924.93 | 13.08 | 1.51  (1.43,1.59) |  |
| Without Metabolic syndrome | Non smoker | 202690 | 10117 | 1813743.34 | 5.58 | 1(Ref.) | 0.5007 | 4493 | 1832453.93 | 2.45 | 1(Ref.) | 0.5176 | 6253 | 1825655.37 | 3.43 | 1(Ref.) | 0.4376 | 15378 | 1845642.18 | 8.33 | 1(Ref.) | 0.4592 |
|  | Smoking group 1 | 7780 | 143 | 71572.19 | 2 | 1.19  (1.01,1.41) |  | 77 | 71781.57 | 1.0727 | 1.3  (1.03,1.63) |  | 73 | 71763.44 | 1.02 | 1.1  (0.87,1.38) |  | 181 | 71986.38 | 2.51 | 1.86  (1.61,2.16) |  |
|  | Smoking group 2 | 25742 | 1407 | 227745.47 | 6.18 | 1.47  (1.39,1.56) |  | 663 | 230272.14 | 2.8792 | 1.52  (1.4,1.66) |  | 829 | 229529.23 | 3.61 | 1.43  (1.33,1.54) |  | 2121 | 232204.1 | 9.13 | 1.65  (1.57,1.72) |  |
|  | Smoking group 3 | 6085 | 526 | 51878.13 | 10.14 | 1.84  (1.68,2.02) |  | 269 | 52779.72 | 5.09665 | 2.13  (1.88,2.41) |  | 298 | 52696.95 | 5.66 | 1.66  (1.48,1.87) |  | 796 | 53665.75 | 14.83 | 1.89  (1.75,2.03) |  |
|  | Smoking group 4 | 17701 | 1818 | 148236.47 | 12.26 | 1.7  (1.61,1.79) |  | 839 | 151810.64 | 5.52662 | 1.79  (1.66,1.94) |  | 1122 | 150572.19 | 7.45 | 1.65  (1.54,1.77) |  | 2876 | 154432.68 | 18.62 | 1.63  (1.56,1.7) |  |
| With  Metabolic syndrome | Non smoker | 161823 | 17714 | 1383184.62 | 12.81 | 1(Ref.) |  | 8057 | 1417209.15 | 5.68512 | 1(Ref.) |  | 11017 | 1405647.43 | 7.84 | 1(Ref.) |  | 23117 | 1442489.82 | 16.03 | 1(Ref.) |  |
|  | Smoking group 1 | 2430 | 116 | 21695.96 | 5.35 | 1.37  (1.14,1.65) |  | 59 | 21885.57 | 2.69584 | 1.4  (1.08,1.81) |  | 65 | 21878.11 | 2.97 | 1.35  (1.05,1.72) |  | 148 | 22087.28 | 6.7 | 2.03  (1.73,2.39) |  |
|  | Smoking group 2 | 16859 | 1757 | 143535.58 | 12.24 | 1.5  (1.42,1.58) |  | 853 | 146792.07 | 5.81094 | 1.54  (1.43,1.66) |  | 1040 | 146174.4 | 7.11 | 1.46  (1.37,1.56) |  | 2091 | 149754.88 | 13.96 | 1.56  (1.49,1.63) |  |
|  | Smoking group 3 | 5573 | 748 | 45902.49 | 16.3 | 1.83  (1.7,1.98) |  | 380 | 47266.55 | 8.03951 | 2  (1.8,2.23) |  | 448 | 46985.17 | 9.53 | 1.78  (1.61,1.96) |  | 925 | 48511.74 | 19.07 | 1.91  (1.78,2.04) |  |
|  | Smoking group 4 | 18055 | 2589 | 146917.37 | 17.62 | 1.63  (1.55,1.7) |  | 1224 | 152132.15 | 8.04564 | 1.66  (1.55,1.77) |  | 1566 | 150604.82 | 10.4 | 1.58  (1.49,1.67) |  | 3340 | 156255.23 | 21.38 | 1.63  (1.57,1.7) |  |
| Non drinker | Non smoker | 282552 | 23710 | 2459339.19 | 9.64 | 1(Ref.) | 0.0002 | 10776 | 2503838.89 | 4.3 | 1(Ref.) | 0.0336 | 14666 | 2488825.91 | 5.89 | 1(Ref.) | 0.0043 | 33596 | 2536917.65 | 13.24 | 1(Ref.) | 0.1835 |
|  | Smoking group 1 | 2022 | 95 | 18046.12 | 5.26 | 1.65  (1.35,2.02) |  | 49 | 18183.27 | 2.69 | 1.61  (1.22,2.14) |  | 52 | 18199.27 | 2.86 | 1.62  (1.23,2.13) |  | 134 | 18346.01 | 7.30 | 1.84  (1.56,2.186) |  |
|  | Smoking group 2 | 13888 | 1471 | 116554.02 | 12.62 | 1.58  (1.5,1.67) |  | 724 | 119140.83 | 6.08 | 1.61  (1.49,1.74) |  | 872 | 118643.95 | 7.35 | 1.56  (1.46,1.68) |  | 2141 | 121495.35 | 17.62 | 1.58  (1.51,1.65) |  |
|  | Smoking group 3 | 3421 | 462 | 27499.57 | 16.8 | 1.78  (1.62,1.95) |  | 258 | 28204.43 | 9.15 | 2.04  (1.79,2.31) |  | 261 | 28226.59 | 9.25 | 1.68  (1.48,1.91) |  | 729 | 29033.94 | 25.11 | 1.88  (1.74,2.03) |  |
|  | Smoking group 4 | 12167 | 1807 | 96676.47 | 18.69 | 1.64  (1.56,1.73) |  | 921 | 99817.65 | 9.23 | 1.74  (1.62,1.873 |  | 1029 | 99527.55 | 10.34 | 1.53  (1.43,1.63) |  | 2799 | 102973.98 | 27.18 | 1.57  (1.51,1.64) |  |
| Mild drinker | Non smoker | 75362 | 3616 | 679912.48 | 5.32 | 1(Ref.) |  | 1572 | 687096.96 | 2.29 | 1(Ref.) |  | 2269 | 684261.56 | 3.32 | 1(Ref.) |  | 4198 | 691879.24 | 6.07 | 1(Ref.) |  |
|  | Smoking group 1 | 6084 | 113 | 55934.63 | 2.02 | 1.04  (0.86,1.26) |  | 57 | 56134.27 | 1.02 | 1.05  (0.8,1.37) |  | 63 | 56092.01 | 1.12 | 1.06  (0.82,1.36) |  | 137 | 56311.99 | 2.43 | 1.96  (1.66,2.33) |  |
|  | Smoking group 2 | 24071 | 1434 | 213713.37 | 6.71 | 1.43  (1.35,1.53) |  | 687 | 216392.98 | 3.17 | 1.49  (1.36,1.64) |  | 826 | 215756.28 | 3.83 | 1.37  (1.27,1.49) |  | 1677 | 218608.73 | 7.67 | 1.66  (1.57,1.76) |  |
|  | Smoking group 3 | 4901 | 476 | 41984.91 | 11.34 | 1.86  (1.69,2.05) |  | 235 | 42880.84 | 5.48 | 2  (1.74,2.3) |  | 276 | 42718.14 | 6.46 | 1.75  (1.54,1.98) |  | 542 | 43683.95 | 12.41 | 1.89  (1.73,2.07) |  |
|  | Smoking group 4 | 16787 | 1877 | 141506.89 | 13.26 | 1.69  (1.59,1.79) |  | 856 | 145543.39 | 5.88 | 1.71  (1.57,1.87) |  | 1169 | 143848.6 | 8.13 | 1.68  (1.56,1.81) |  | 2374 | 148210.15 | 16.02 | 1.73  (1.64,1.82) |  |
| Heavy drinker | Non smoker | 6599 | 505 | 57676.28 | 8.76 | 1(Ref.) |  | 202 | 58727.23 | 3.44 | 1(Ref.) |  | 335 | 58215.33 | 5.75 | 1(Ref.) |  | 701 | 59335.11 | 11.81 | 1(Ref.) |  |
|  | Smoking group 1 | 2104 | 51 | 19287.4 | 2.64 | 1.19  (0.89,1.59) |  | 30 | 19349.6 | 1.55 | 1.52  (1.03,2.23) |  | 23 | 19350.27 | 1.19 | 0.94  (0.62,1.43) |  | 58 | 19415.67 | 2.99 | 2.22  (1.7,2.91) |  |
|  | Smoking group 2 | 4642 | 259 | 41013.65 | 6.32 | 1.18  (1.01,1.37) |  | 105 | 41530.41 | 2.53 | 1.14  (0.9,1.44) |  | 171 | 41303.41 | 4.14 | 1.23  (1.02,1.48) |  | 394 | 41854.91 | 9.41 | 1.62  (1.43,1.83) |  |
|  | Smoking group 3 | 3336 | 336 | 28296.14 | 11.87 | 1.74  (1.51,1.99) |  | 156 | 28961.01 | 5.39 | 1.92  (1.56,2.37) |  | 209 | 28737.4 | 7.27 | 1.68  (1.41,2) |  | 450 | 29459.59 | 15.28 | 2  (1.77,2.25) |  |
|  | Smoking group 4 | 6802 | 723 | 56970.49 | 12.69 | 1.48  (1.32,1.66) |  | 286 | 58581.75 | 4.88 | 1.42  (1.19,1.7) |  | 490 | 57800.86 | 8.48 | 1.53  (1.33,1.76) |  | 1043 | 59503.79 | 17.53 | 1.64  (1.49,1.81) |  |
| Without  Regular exercise | Non smoker | 295111 | 23357 | 2576774.74 | 9.06 | 1(Ref.) | 0.5681 | 10543 | 2621028.01 | 4.02 | 1(Ref.) | 0.4394 | 14508 | 2605335.7 | 5.57 | 1(Ref.) | 0.5182 | 33320 | 2653082.17 | 12.56 | 1(Ref.) | <.0001 |
|  | Smoking group 1 | 8514 | 225 | 77641.56 | 2.9 | 1.29  (1.13,1.48) |  | 113 | 77994.41 | 1.45 | 1.3  (1.07,1.57) |  | 124 | 77963.1 | 1.59 | 1.27  (1.06,1.52) |  | 278 | 78334.65 | 3.55 | 1.87  (1.66,2.1) |  |
|  | Smoking group 2 | 34217 | 2573 | 297619.44 | 8.65 | 1.47  (1.41,1.54) |  | 1232 | 302361.48 | 4.07 | 1.51  (1.42,1.61) |  | 1527 | 301177.41 | 5.07 | 1.44  (1.36,1.53) |  | 3452 | 306310.2 | 11.27 | 1.55  (1.49,1.61) |  |
|  | Smoking group 3 | 9775 | 1070 | 81900.15 | 13.06 | 1.82  (1.7,1.94) |  | 542 | 83861.74 | 6.46 | 2.01  (1.83,2.2) |  | 636 | 83450.92 | 7.62 | 1.74  (1.6,1.9) |  | 1444 | 85615.3 | 16.87 | 1.84  (1.74,1.94) |  |
|  | Smoking group 4 | 29471 | 3679 | 242553.21 | 15.17 | 1.65  (1.58,1.71) |  | 1720 | 249884.69 | 6.88 | 1.69  (1.6,1.79) |  | 2241 | 247620.08 | 9.05 | 1.6  (1.52,1.68) |  | 5202 | 255529.74 | 20.358 | 1.58  (1.53,1.63) |  |
| With  Regular exercise | Non smoker | 69402 | 4474 | 620153.22 | 7.21 | 1(Ref.) |  | 2007 | 628635.07 | 3.19 | 1(Ref.) |  | 2762 | 625967.1 | 4.41 | 1(Ref.) |  | 5175 | 635049.83 | 8.15 | 1(Ref.) |  |
|  | Smoking group 1 | 1696 | 34 | 15626.6 | 2.18 | 1.11  (0.79,1.56) |  | 23 | 15672.73 | 1.47 | 1.55  (1.02,2.34) |  | 14 | 15678.45 | 0.89 | 0.81  (0.48,1.38) |  | 51 | 15739.02 | 3.24 | 2.35  (1.79,3.1) |  |
|  | Smoking group 2 | 8384 | 591 | 73661.61 | 8.02 | 1.55  (1.42,1.69) |  | 284 | 74702.73 | 3.8 | 1.63  (1.43,1.85) |  | 342 | 74526.23 | 4.59 | 1.47  (1.31,1.65) |  | 760 | 75648.78 | 10.05 | 1.89  (1.75,2.04) |  |
|  | Smoking group 3 | 1883 | 204 | 15880.48 | 12.85 | 1.96  (1.7,2.26) |  | 107 | 16184.53 | 6.61 | 2.32  (1.91,2.83) |  | 110 | 16231.21 | 6.78 | 1.67  (1.37,2.02) |  | 277 | 16562.19 | 16.72 | 2.25  (1.99,2.54) |  |
|  | Smoking group 4 | 6285 | 728 | 52600.63 | 13.84 | 1.7  (1.57,1.84) |  | 343 | 54058.1 | 6.35 | 1.8  (1.61,2.03) |  | 447 | 53556.93 | 8.35 | 1.65  (1.49,1.83) |  | 1014 | 55158.17 | 18.38 | 1.92  (1.79,2.05) |  |
| eGFR < 60 | Non smoker | 114947 | 5664 | 1036387.49 | 5.47 | 1(Ref.) | <.0001 | 2501 | 1047818.56 | 2.39 | 1(Ref.) | 0.0002 | 3532 | 1043828.33 | 3.38 | 1(Ref.) | 0.0002 | 6368 | 1056096.5 | 6.03 | 1(Ref.) | <.0001 |
|  | Smoking group 1 | 7758 | 142 | 71440.97 | 1.99 | 1.18  (1,1.4) |  | 84 | 71628.05 | 1.17 | 1.43  (1.15,1.78) |  | 67 | 71679.57 | 0.93 | 1  (0.79,1.28) |  | 159 | 71893.41 | 2.21 | 2.48  (2.12,2.91) |  |
|  | Smoking group 2 | 22430 | 1291 | 199487.7 | 6.47 | 1.57  (1.48,1.67) |  | 645 | 201828.58 | 3.2 | 1.73  (1.58,1.9) |  | 716 | 201524.66 | 3.55 | 1.43  (1.32,1.56) |  | 1419 | 204025.09 | 6.96 | 1.82  (1.71,1.93) |  |
|  | Smoking group 3 | 6417 | 612 | 54914.26 | 11.14 | 2.02  (1.85,2.2) |  | 313 | 56022.05 | 5.59 | 2.35  (2.08,2.66) |  | 351 | 55867.44 | 6.28 | 1.84  (1.64,2.06) |  | 732 | 57064.81 | 12.8 | 2.29  (2.12,2.47) |  |
|  | Smoking group 4 | 16788 | 1851 | 141454.97 | 13.09 | 1.83  (1.73,1.94) |  | 845 | 145397.35 | 5.81 | 1.92  (1.77,2.08) |  | 1141 | 143953.03 | 7.93 | 1.77  (1.65,1.9) |  | 2264 | 148176.03 | 15.3 | 1.89  (1.8,1.99) |  |
| eGFR ≥ 60 | Non smoker | 249566 | 22167 | 2160540.47 | 10.26 | 1(Ref.) |  | 10049 | 2201844.53 | 4.56 | 1(Ref.) |  | 13738 | 2187474.47 | 6.28 | 1(Ref.) |  | 32127 | 2232035.5 | 14.4 | 1(Ref.) |  |
|  | Smoking group 1 | 2452 | 117 | 21827.18 | 5.36 | 1.44  (1.2,1.73) |  | 52 | 22039.09 | 2.36 | 1.29  (0.98,1.7) |  | 71 | 21961.98 | 3.23 | 1.51  (1.2,1.91) |  | 170 | 22180.25 | 7.66 | 1.65  (1.42,1.92) |  |
|  | Smoking group 2 | 20171 | 1873 | 171793.35 | 10.9 | 1.45  (1.38,1.52) |  | 871 | 175235.64 | 4.97 | 1.44  (1.34,1.55) |  | 1153 | 174178.97 | 6.62 | 1.47  (1.38,1.56) |  | 2793 | 177933.89 | 15.7 | 1.53  (1.47,1.6) |  |
|  | Smoking group 3 | 5241 | 662 | 42866.36 | 15.44 | 1.73  (1.6,1.88) |  | 336 | 44024.22 | 7.63 | 1.89  (1.69,2.12) |  | 395 | 43814.69 | 9.02 | 1.67  (1.5,1.85) |  | 989 | 45112.68 | 21.9 | 1.73  (1.62,1.84) |  |
|  | Smoking group 4 | 18968 | 2556 | 153698.87 | 16.63 | 1.57  (1.5,1.64) |  | 1218 | 158545.44 | 7.68 | 1.62  (1.52,1.73) |  | 1547 | 157223.97 | 9.84 | 1.52  (1.43,1.6) |  | 3952 | 162511.88 | 24.31 | 1.54  (1.48,1.59) |  |
| Without  Diabetes | Non smoker | 290468 | 17508 | 2588104.93 | 6.76 | 1(Ref.) | 0.0026 | 7768 | 2621497.05 | 2.96 | 1(Ref.) | 0.0013 | 10811 | 2609877.43 | 4.14 | 1(Ref.) | 0.0399 | 23903 | 2645523.58 | 9.04 | 1(Ref.) | 0.7807 |
|  | Smoking group 1 | 9090 | 184 | 83517.33 | 2.2 | 1.26  (1.09,1.46) |  | 105 | 83765.87 | 1.25 | 1.45  (1.19,1.77) |  | 89 | 83827.33 | 1.06 | 1.1  (0.89,1.36) |  | 216 | 84097.12 | 2.57 | 1.92  (1.68,2.2) |  |
|  | Smoking group 2 | 32919 | 1883 | 291972.28 | 6.45 | 1.46  (1.38,1.53) |  | 920 | 295296.2 | 3.12 | 1.56  (1.45,1.67) |  | 1082 | 294635.04 | 3.67 | 1.39  (1.3,1.48) |  | 2535 | 298214.62 | 8.5 | 1.6  (1.53,1.67) |  |
|  | Smoking group 3 | 7871 | 699 | 67560.7 | 10.35 | 1.89  (1.75,2.04) |  | 355 | 68848.34 | 5.16 | 2.14  (1.91,2.39) |  | 389 | 68717.2 | 5.66 | 1.7  (1.53,1.89) |  | 907 | 70080.58 | 12.94 | 1.84  (1.72,1.97) |  |
|  | Smoking group 4 | 23577 | 2449 | 198993.02 | 12.31 | 1.7  (1.63,1.79) |  | 1159 | 203836.34 | 5.69 | 1.82  (1.7,1.95) |  | 1459 | 202560.52 | 7.2 | 1.62  (1.52,1.72) |  | 3431 | 207734.08 | 16.52 | 1.62  (1.56,1.68) |  |
| With  Diabetes | Non smoker | 74045 | 10323 | 608823.02 | 16.96 | 1(Ref.) |  | 4782 | 628166.04 | 7.61 | 1(Ref.) |  | 6459 | 621425.37 | 10.39 | 1(Ref.) |  | 14592 | 642608.42 | 22.71 | 1(Ref.) |  |
|  | Smoking group 1 | 1120 | 75 | 9750.82 | 7.69 | 1.39  (1.11,1.75) |  | 31 | 9901.27 | 3.13 | 1.15(0.81,1.64) |  | 49 | 9814.22 | 4.99 | 1.58  (1.19,2.1) |  | 113 | 9976.55 | 11.33 | 2.16  (1.79,2.6) |  |
|  | Smoking group 2 | 9682 | 1281 | 79308.76 | 16.15 | 1.52  (1.43,1.61) |  | 596 | 81768.01 | 7.29 | 1.48  (1.35,1.62) |  | 787 | 81068.59 | 9.71 | 1.52  (1.41,1.65) |  | 1677 | 83744.36 | 20.03 | 1.61  (1.53,1.69) |  |
|  | Smoking group 3 | 3787 | 575 | 30219.92 | 19.03 | 1.7  (1.56,1.85) |  | 294 | 31197.93 | 9.42 | 1.86  (1.65,2.11) |  | 357 | 30964.92 | 11.53 | 1.69  (1.51,1.89) |  | 814 | 32096.9 | 25.36 | 1.9  (1.77,2.04) |  |
|  | Smoking group 4 | 12179 | 1958 | 96160.82 | 20.36 | 1.53  (1.45,1.61) |  | 904 | 100106.45 | 9.03 | 1.51  (1.4,1.63) |  | 1229 | 98616.48 | 12.46 | 1.53  (1.44,1.64) |  | 2785 | 102953.83 | 27.05 | 1.6  (1.53,1.67) |  |
| Without  Hypertension | Non smoker | 173565 | 6515 | 1577727.81 | 4.13 | 1(Ref.) | <.0001 | 3059 | 1589583.01 | 1.92 | 1(Ref.) | 0.0001 | 3809 | 1586437.71 | 2.4 | 1(Ref.) | <.0001 | 8358 | 1599101.44 | 5.23 | 1(Ref.) | 0.0067 |
|  | Smoking group 1 | 7630 | 116 | 70515.19 | 1.65 | 1.14  (0.95,1.37) |  | 72 | 70665.66 | 1.02 | 1.39  (1.1,1.76) |  | 48 | 70714.58 | 0.68 | 0.88  (0.66,1.18) |  | 124 | 70875.6 | 1.75 | 1.84  (1.54,2.2) |  |
|  | Smoking group 2 | 23437 | 1049 | 210896.84 | 4.97 | 1.54  (1.44,1.65) |  | 550 | 212578.57 | 2.59 | 1.68  (1.53,1.85) |  | 558 | 212574.37 | 2.63 | 1.43  (1.3,1.56) |  | 1256 | 214371.23 | 5.86 | 1.63  (1.54,1.74) |  |
|  | Smoking group 3 | 5658 | 408 | 49262.55 | 8.28 | 1.94  (1.75,2.15) |  | 224 | 49947.33 | 4.48 | 2.28  (1.98,2.62 |  | 218 | 49942.7 | 4.37 | 1.76  (1.53,2.02) |  | 567 | 50688.67 | 11.19 | 2.1  (1.92,2.28) |  |
|  | Smoking group 4 | 15189 | 1389 | 130241.77 | 10.66 | 1.93  (1.82,2.05) |  | 680 | 132906.2 | 5.12 | 2.03  (1.86,2.22) |  | 810 | 132329.6 | 6.12 | 1.87  (1.73,2.03) |  | 1851 | 135193.72 | 13.69 | 1.76  (1.67,1.85) |  |
| With  Hypertension | Non smoker | 190948 | 21316 | 1619200.15 | 13.16 | 1(Ref.) |  | 9491 | 1660080.07 | 5.72 | 1(Ref.) |  | 13461 | 1644865.09 | 8.18 | 1(Ref.) |  | 30137 | 1689030.56 | 17.84 | 1(Ref.) |  |
|  | Smoking group 1 | 2580 | 143 | 22752.96 | 6.28 | 1.45  (1.23,1.71) |  | 64 | 23001.48 | 2.78 | 1.35  (1.05,1.73) |  | 90 | 22926.98 | 3.93 | 1.56  (1.26,1.92) |  | 205 | 23198.06 | 8.84 | 2.06  (1.8,2.37) |  |
|  | Smoking group 2 | 19164 | 2115 | 160384.21 | 13.19 | 1.49  (1.42,1.56) |  | 966 | 164485.64 | 5.87 | 1.49  (1.38,1.59) |  | 1311 | 163129.26 | 8.04 | 1.48  (1.4,1.57) |  | 2956 | 167587.75 | 17.64 | 1.61  (1.55,1.68) |  |
|  | Smoking group 3 | 6000 | 866 | 48518.07 | 17.85 | 1.84  (1.72,1.98) |  | 425 | 50098.94 | 8.48 | 2.01  (1.81,2.22) |  | 528 | 49739.42 | 10.62 | 1.77  (1.62,1.94) |  | 1154 | 51488.81 | 22.41 | 1.86  (1.75,1.97) |  |
|  | Smoking group 4 | 20567 | 3018 | 164912.07 | 18.3 | 1.59  (1.52,1.65) |  | 1383 | 171036.59 | 8.09 | 1.62  (1.52,1.72) |  | 1878 | 168847.4 | 11.12 | 1.55  (1.46,1.63) |  | 4365 | 175494.19 | 24.87 | 1.61  (1.55,1.66) |  |

^a^ Multivariate model 2 was adjusted for age, sex, income status, alcohol consumption, regular physical activity, BMI, eGFR, proteinuria and metabolic syndrome.

CCVDs, cardio-cerebrovascular diseases; MI, myocardial infarction; IR, incidence rate; BMI, body mass index; eGFR, estimated glomerular filtration rate

**Supplementary Table 5. Risk by stratified analysis of smoking intensity and initiation age.**

|  | Exposure | CCVDs | MI | Stroke | Death |
| --- | --- | --- | --- | --- | --- |
| Age ≥65 or <65 | Non smoker | 1(Ref.) | 1(Ref.) | 1(Ref.) | 1(Ref.) |
|  | Smoking group 1 | 1.25(1.102,1.417) | 1.363(1.145,1.623) | 1.163(0.98,1.381) | 1.963(1.759,2.192) |
|  | Smoking group 2 | 1.486(1.427,1.547) | 1.533(1.446,1.625) | 1.448(1.375,1.526) | 1.599(1.546,1.655) |
|  | Smoking group 3 | 1.84(1.732,1.954) | 2.051(1.884,2.233) | 1.735(1.604,1.877) | 1.896(1.802,1.996) |
|  | Smoking group 4 | 1.658(1.597,1.72) | 1.705(1.615,1.8) | 1.613(1.539,1.691) | 1.627(1.577,1.677) |
| Sex | Non smoker | 1(Ref.) | 1(Ref.) | 1(Ref.) | 1(Ref.) |
|  | Smoking group 1 | 1.266(1.117,1.435) | 1.335(1.122,1.589) | 1.203(1.014,1.427) | 1.934(1.733,2.159) |
|  | Smoking group 2 | 1.486(1.427,1.547) | 1.533(1.446,1.626) | 1.448(1.374,1.525) | 1.601(1.547,1.656) |
|  | Smoking group 3 | 1.837(1.73,1.951) | 2.054(1.886,2.236) | 1.73(1.6,1.871) | 1.895(1.801,1.995) |
|  | Smoking group 4 | 1.654(1.594,1.717) | 1.71(1.62,1.806) | 1.606(1.532,1.684) | 1.628(1.579,1.679) |
| Obesity* | Non smoker | 1(Ref.) | 1(Ref.) | 1(Ref.) | 1(Ref.) |
|  | Smoking group 1 | 1.269(1.12,1.438) | 1.337(1.124,1.591) | 1.201(1.012,1.425) | 1.929(1.728,2.154) |
|  | Smoking group 2 | 1.486(1.427,1.547) | 1.533(1.446,1.626) | 1.447(1.374,1.524) | 1.598(1.544,1.654) |
|  | Smoking group 3 | 1.838(1.73,1.952) | 2.054(1.887,2.237) | 1.73(1.6,1.871) | 1.894(1.8,1.994) |
|  | Smoking group 4 | 1.655(1.594,1.717) | 1.71(1.62,1.806) | 1.606(1.532,1.684) | 1.627(1.578,1.677) |
| Metabolic syndrome | Non smoker | 1(Ref.) | 1(Ref.) | 1(Ref.) | 1(Ref.) |
|  | Smoking group 1 | 1.266(1.117,1.435) | 1.335(1.122,1.589) | 1.205(1.016,1.43) | 1.934(1.732,2.159) |
|  | Smoking group 2 | 1.486(1.427,1.547) | 1.534(1.446,1.626) | 1.448(1.374,1.525) | 1.6(1.547,1.656) |
|  | Smoking group 3 | 1.838(1.731,1.952) | 2.055(1.887,2.237) | 1.731(1.6,1.872) | 1.896(1.802,1.996) |
|  | Smoking group 4 | 1.655(1.595,1.717) | 1.71(1.62,1.806) | 1.606(1.532,1.684) | 1.628(1.579,1.679) |
| Drinking | Non smoker | 1(Ref.) | 1(Ref.) | 1(Ref.) | 1(Ref.) |
|  | Smoking group 1 | 1.266(1.117,1.435) | 1.334(1.121,1.587) | 1.206(1.017,1.431) | 1.934(1.732,2.159) |
|  | Smoking group 2 | 1.485(1.427,1.547) | 1.533(1.445,1.625) | 1.447(1.374,1.524) | 1.601(1.547,1.656) |
|  | Smoking group 3 | 1.838(1.731,1.952) | 2.055(1.888,2.237) | 1.731(1.6,1.872) | 1.896(1.801,1.995) |
|  | Smoking group 4 | 1.655(1.595,1.717) | 1.711(1.62,1.806) | 1.606(1.531,1.684) | 1.628(1.579,1.679) |
| Regular exercise | Non smoker | 1(Ref.) | 1(Ref.) | 1(Ref.) | 1(Ref.) |
|  | Smoking group 1 | 1.269(1.12,1.438) | 1.337(1.124,1.59) | 1.202(1.013,1.426) | 1.934(1.732,2.159) |
|  | Smoking group 2 | 1.486(1.427,1.547) | 1.533(1.446,1.626) | 1.447(1.374,1.525) | 1.601(1.547,1.656) |
|  | Smoking group 3 | 1.838(1.73,1.952) | 2.055(1.887,2.237) | 1.73(1.6,1.871) | 1.897(1.802,1.996) |
|  | Smoking group 4 | 1.655(1.594,1.717) | 1.71(1.62,1.806) | 1.606(1.532,1.684) | 1.628(1.579,1.679) |
| GFR ≥60 or <60 | Non smoker | 1(Ref.) | 1(Ref.) | 1(Ref.) | 1(Ref.) |
|  | Smoking group 1 | 1.268(1.118,1.437) | 1.336(1.123,1.59) | 1.207(1.017,1.431) | 1.941(1.739,2.166) |
|  | Smoking group 2 | 1.488(1.429,1.549) | 1.535(1.448,1.628) | 1.449(1.376,1.527) | 1.602(1.548,1.657) |
|  | Smoking group 3 | 1.841(1.733,1.955) | 2.058(1.89,2.24) | 1.733(1.603,1.875) | 1.896(1.802,1.996) |
|  | Smoking group 4 | 1.656(1.596,1.718) | 1.712(1.621,1.807) | 1.607(1.533,1.685) | 1.628(1.578,1.678) |
| Diabetes | Non smoker | 1(Ref.) | 1(Ref.) | 1(Ref.) | 1(Ref.) |
|  | Smoking group 1 | 1.298(1.145,1.471) | 1.367(1.149,1.626) | 1.24(1.045,1.471) | 1.997(1.789,2.229) |
|  | Smoking group 2 | 1.48(1.422,1.541) | 1.526(1.439,1.618) | 1.443(1.37,1.52) | 1.602(1.548,1.658) |
|  | Smoking group 3 | 1.799(1.694,1.911) | 2.01(1.846,2.188) | 1.694(1.566,1.832) | 1.868(1.774,1.966) |
|  | Smoking group 4 | 1.624(1.565,1.686) | 1.677(1.588,1.77) | 1.577(1.504,1.654) | 1.608(1.559,1.658) |
| Hypertension | Non smoker | 1(Ref.) | 1(Ref.) | 1(Ref.) | 1(Ref.) |
|  | Smoking group 1 | 1.287(1.135,1.459) | 1.352(1.136,1.608) | 1.23(1.037,1.46) | 1.968(1.763,2.197) |
|  | Smoking group 2 | 1.501(1.442,1.563) | 1.546(1.458,1.64) | 1.465(1.39,1.543) | 1.615(1.561,1.671) |
|  | Smoking group 3 | 1.873(1.763,1.989) | 2.086(1.916,2.271) | 1.767(1.633,1.911) | 1.926(1.83,2.027) |
|  | Smoking group 4 | 1.679(1.618,1.742) | 1.731(1.64,1.828) | 1.632(1.556,1.711) | 1.646(1.596,1.698) |

^a^ Multivariate model 2 was adjusted for age, sex, income status, alcohol consumption, regular physical activity, BMI, eGFR, proteinuria and metabolic syndrome.

*obesity status was based on a BMI threshold of 25 kg/m².

CCVDs, cardio-cerebrovascular diseases; MI, myocardial infarction.
